# A network meta-analysis of the effect of physical exercise on core symptoms in patients with autism spectrum disorders

**DOI:** 10.3389/fneur.2024.1360434

**Published:** 2024-05-09

**Authors:** Lili Li, Shuqi Jia, Peng Wang, Shufan Li, Xing Wang, Xiaoyi Zhu

**Affiliations:** ^1^Sports Department, Shanghai University of Engineering Science, Shanghai, China; ^2^School of Physical Education, Shanghai University of Sport, Shanghai, China; ^3^Guangxi Health Science College, Nanning, Guangxi Zhuang Autonomous Region, China

**Keywords:** physical activity, autism spectrum disorder, motor intervention, core symptoms, network meta-analysis

## Abstract

**Objective:**

To compare the effects of various sports exercise programs on the core symptoms of patients with autism spectrum disorder (ASD).

**Methods:**

We searched the China National Knowledge Infrastructure, VIP databases, Wanfang databases, Cochrane Library, PubMed, EMBASE, and Web of Science databases from their inception to February 2023 for randomized controlled trial that investigated the effect of sports exercise on the core symptoms of ASD. The overall risk of bias in the included literature was summarized using the revised Cochrane Randomized Trial Risk of Bias Tool (ROB2), and network meta-analysis was used to compare the intervention effects.

**Results:**

A total of 30 studies involving 1,375 participants were included. The results showed that sports exercise programs, including 8–12 weeks of ball sports (SMD = −5.35, 95%CI: −7.57, −3.23), horse riding (SMD = −3.71, 95%CI: −6.18, −1.13), 8–12 weeks of comprehensive sports exercise (SMD = −2.17, 95%CI: −3.99, −0.44), and more than 12 weeks of comprehensive sports exercise (SMD = −3.75, 95%CI: −6.33, −1.24), significantly improved social interaction disorders. Furthermore, 8–12 weeks of ball sports (SMD = −4.36, 95%CI: 2.04, 6.73) and more than 12 weeks of comprehensive sports exercise (SMD = 3.65, 95%CI: 1.40, 6.08) significantly improved repetitive behaviors and restricted interests.

**Conclusion:**

Sports exercise can improve the core symptoms of ASD patients, and different symptoms show a selective response to different exercise elements.

**Systematic review registration:**

https://www.crd.york.ac.uk/prospero/, identifier CRD42023455806.

## 1 Introduction

Autistic spectrum disorder (ASD) is an early onset neurodevelopmental disorder (1), with social communication and interaction disorders, and the repetition of the stereotyped interest behavior as the core symptoms. These abnormal manifestations seriously affect children’s survival, development, and the participation in daily activities at home, on campus and in the community (2), which brings a heavy burden to patients and families. In recent years, the global incidence of ASD has been on the rise, and the overall prevalence rate in developed countries is about 1.5%. According to the statistics of the Centers for Disease Control and Prevention in 2021, the prevalence rate of ASD in children aged from 0 to 8 years old is 2.47% (3). According to China’s clinical diagnostic criteria, the prevalence rate of ASD in China also reaches 39.23 in every 10,000 people (4).

A sedentary lifestyle not only affects children’s physical health, but also may isolate and deprive children of their social adaptive functions and skills (5). Exercise intervention can improve the overall symptoms of autistic patients by 37% (6), especially in behavioral and academic aspects. Studies have found that jogging, roller skating, hydrotherapy exercises and sports games can reduce the frequency of stereotypical behaviors (7). What’s more, physical exercise can have a positive impact on the cognition, behavior, emotion and communication of children with ASD in a relatively short time (8, 9).

Physical exercise can improve the survival status of patients with autism spectrum disorders, and regular physical exercise is an effective way to improve social interaction disorders and repetitive behavior symptoms in patients with ASD, which has been publicly established. However, in practice, we increasingly feel that exercise is composed of five elements: exercise form, cycle, frequency, intensity and duration. Which factor has a greater impact on the outcome index? Can the comprehensive effects of these five factors be comprehensively considered? The solution of these problems is of great significance to find the best exercise plan. In this study, the Bayesian mesh meta model was used to combine the results of direct comparison and indirect comparison to conduct pin-to-pair comparison and quantitative ranking of various intervention measures. Moreover, from the perspective of movement element combination, the intervention effects of various exercise programs on the core symptoms of patients with autism spectrum disorder were compared, and the optimal exercise program was finally formed to provide low-cost, practical and accurate exercise intervention evidence for the rehabilitation of patients with autism spectrum disorders.

## 2 Research methods

This systematic review was prospectively registered with the National Institute for Health Research website PROSPERO. Details of the protocol can be accessed at: https://www.crd.york.ac.uk/prospero/, identifier CRD42023455806.

### 2.1 Literature sources

We collated and made statistics of the included literature according to the requirements of the International Guidelines for Writing Systematic Reviews. What’s more, the study followed the PRISMA statement (Preferred Reporting Items for Systematic Reviews and Meta-analysis) and the requirements of the Cochrane workbook (10).

All relevant literature from PubMed, Web of Science (WOS), EBSCOhost, Cochrane Library, China National Knowledge Infrastructure (CNKI), Wanfang databases, and VIP databases up to February 2023 has been thoroughly searched and reviewed. By combining subject words with free words, the key words “Movement/Physical exercise/Physical activity/exercise/sport/Training, Exercise/Physical Exercises/training/ motion/activity/physical therapy/sport,” “autistic disorder/Autism Spectrum Disorders/Autistic Spectrum Disorders/Disorder, Autistic/Spectrum/Early Infantile Autism/ Disorders, Asperger/Syndrome, Asperger,” “randomized controlled trial/ randomized/controlled/trial/randomized controlled trial/random/random allocation/RCT/RCTs” were searched in PubMed, Web of Science, EBSCOhost and Cochrane Library. The Chinese search terms were consistent with the English search terms and were retrieved from the Chinese databases CNKI, Wanfang and VIP; At the same time, the included literature and related review references were traced to ensure the comprehensiveness of the literature retrieval.

### 2.2 Inclusion and exclusion criteria of the literature

Inclusion criteria: (1) Study type: randomized controlled trial (RCTS); (2) Research objects: Those who have been diagnosed with ASD or those with autism spectrum disorder who met the Diagnostic and Statistical Manual of Mental Disorders-IV or-V criteria for ASD diagnosis (The Fourth Edition of Diagnostic and Statistical Manual of Mental Disorders, DSM-IV or-V); (3) Intervention measures: the control group received routine rehabilitation treatment or had no intervention, and the experimental group added physical exercise on the basis of the control group; (4) Outcome indicators: The outcome indicators or partial outcome indicators were social interaction disorders or the repetition of the stereotyped behaviors.

Exclusion criteria: (1) Review, animal experiments, republished literature, etc.; (2) The literature with unclear description of experimental data, incomplete data, poor quality evaluation or the literature whose original data cannot be gotten or transformed after we contact the author; (3) The subject has other physical diseases; (4) Literature with unclear diagnostic criteria or intervention programs.

### 2.3 Literature screening and data extraction

The literature retrieved was imported into Endnote X9 software for de-duplication, and the titles and abstracts were read for preliminary screening. Two independent researchers would extract data according to the pre-designed table. If there was any disagreement, the third researcher would be invited to discuss and vote together.

Data extraction contents: (1) Basic literature information (first author, publication year); (2) Relevant information of subjects (including number of cases, age, intervention measures, outcome indicators, etc.); (3) Design types and quality evaluation information of literature; (4) Changes and standard deviations of outcome indicators.

### 2.4 Quality evaluation

The included literature was assessed by the investigator for risk of bias according to “*Cochrane Handbook for the Systematic Evaluation of Interventions, version 6.3, 20–22*” (11). Items assessed included (1) bias arising from the randomization process, (2) bias arising from deviations from the intended intervention, (3) bias arising from missing outcome data, (4) bias in outcome measures, and (5) bias in selective reporting of outcomes. The quality of the studies included in each domain was assessed using three options: “high risk,” “low risk” and “some risk.” If all domains were ‘low risk’, the outcome was ‘low risk’ and graded A. If some domains were ‘some risk’ and there was no ‘high risk’, then the outcome was ‘some risk’ and graded B. If one of the domains was ‘high risk’ and there was no ‘high risk’, then the outcome was ‘some risk’ and graded B. If one of the domains is ‘high risk’, then the score is ‘high risk’ If one of the domains is ‘high risk’, then the score is ‘high risk’ and the rating is C. The risk of bias for each sub-dimension was summarized using the Revised Cochrane risk-of-bias tool for randomized trials (ROB2) to obtain the overall risk of bias for the included literature.

### 2.5 Certainty of the evidence

The CINeMA web application (which adapts GRADE domains to network meta-analysis) was used to evaluate confidence in findings from the primary network meta-analyses due to: risk of bias within comparisons, publication bias, indirectness, imprecision, heterogeneity and incoherence (12). A detailed description of the assessment process is provided in Appendix 6 on the eAddenda.

### 2.6 Statistical methods

All outcome indicators in this study were continuous variables. Standardized mean difference (SMD) was adopted as the effect size, in which the absence of 0 in the 95% confidence interval (CI) indicated that the difference was significant.

In traditional meta-analysis, effect size combination, subgroup analysis, heterogeneity test and sensitivity analysis were performed for included studies. The heterogeneity among included studies was analyzed by *I*^2^ and *p*-value test. If *I*^2^ < 50% and *p* > 0.1, the studies were considered homogenous and could be analyzed by fixed-effect model. If *I*^2^ ≥ 50% and *p* < 0.1, the source of heterogeneity should be further determined. After the obvious clinical heterogeneity was excluded, random effects model was used for analysis.

In mesh meta-analysis, R4.2.1 and JAGS 4.3.0 were used for model calculation and verification, and Stata17.0 was used to draw network evidence plots, and publication bias was identified by drawing comparation-correction funnel plots. Among the relationships between intervention measures, the dots represented the intervention methods of physical exercise, the area of dots represented the sample size, and the lines between dots indicated direct comparison between the two exercises. The thicker the lines were, the more studies were conducted. If there was no line between the two intervention measures, it indicated that there was no direct comparison and indirect comparative analysis would be conducted. The consistency model and inconsistent model deviance information criterion (DIC) were used for comparison. If the difference between the two models was less than or equal to 5, it indicated that the consistency premise was basically met. Then the consistency model was used for calculation. Bayes Markov chain-Monte Carlo model was used to compare the intervention effects of different exercise schemes. Four chains were initially set for simulation, in which iteration step size was 1, iteration number was 50,000 times, and the first 20,000 times were used for annealing to eliminate the influence of initial value. The heterogeneity was quantified using *I*^2^ statistics. The potential scale reduced factor (PSRF) was used to evaluate the iterative effect of inter-chain and intra-chain variances. If PSRF was close to or equal to 1, the model convergence was good and the results were stable. Otherwise, the extended operation was continued. What’s more, surface under the cumulative ranking (SUCRA) was used to reflect the cumulative ranking of interventions, where 0 ≤ SUCRA≤100%, and the higher the value, the better the effect.

## 3 Results

### 3.1 Literature search results

**Figure 1:**
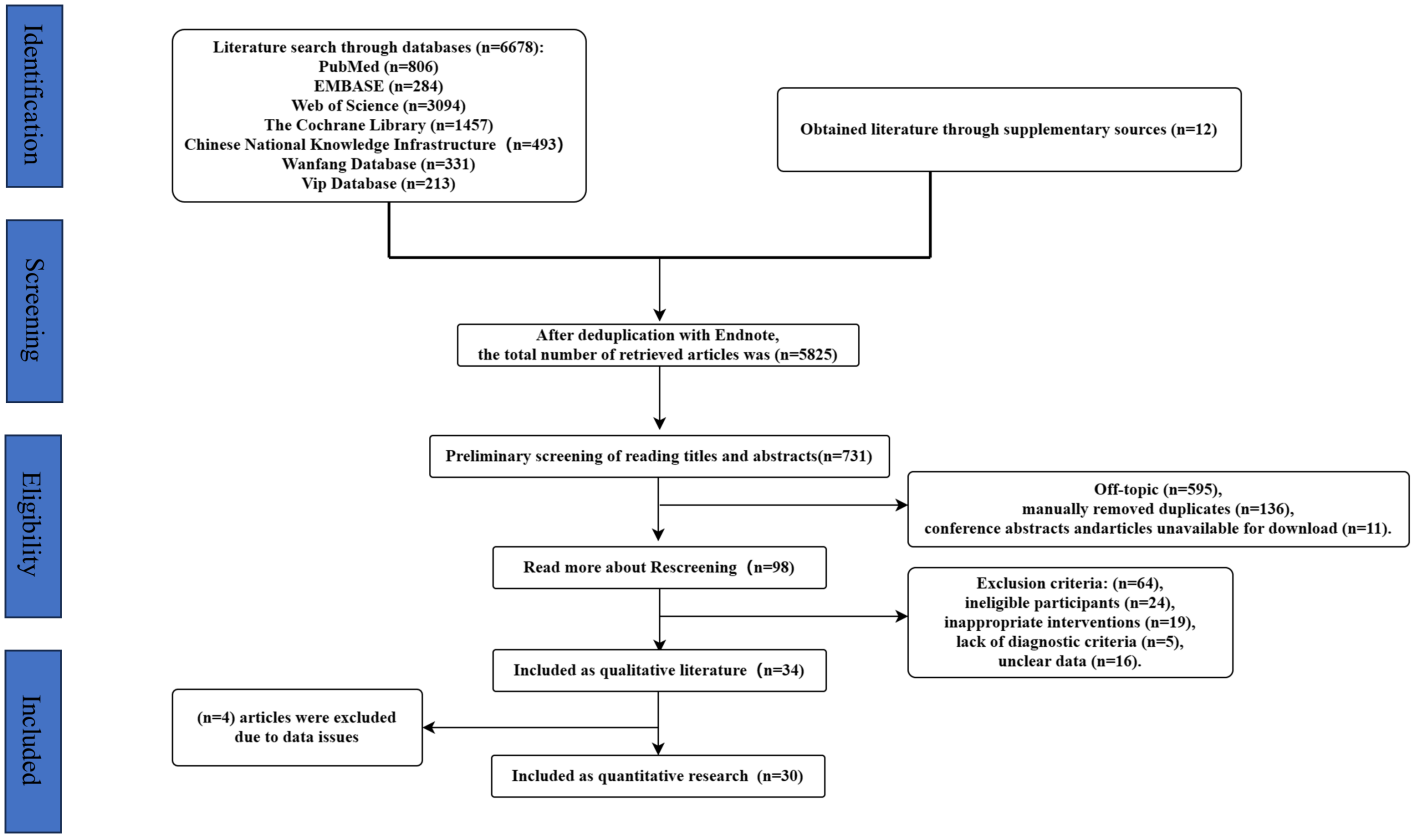
Flow chart of literature selection and inclusion.

A total of 6,678 literature was retrieved from China National Knowledge Network (*n* = 493), Wanfang (*n* = 331), VIP (*n* = 213), PubMed (*n* = 806), Embase (*n* = 284), Cochrane (*n* = 1,457) and Web of Science (*n* = 3,094), 12 literature was manually retrieved. After reading the title and abstract, there were still 731 papers after the initial screening. After reading the full text and re-screening, 98 papers were found to be consistent. After excluding the papers with inconsistent theme, inconsistent object and unclear data, 30 papers were finally included (Figure 1). Two separate independent reviewers (PW and FL) screened the literature during the inclusion process and the Cohen’s kappa value for both researchers was 0.84, which is good agreement.

### 3.2 Basic features of the literature included

Among the 30 literature included (The inclusion of this paper found 14 articles from China, 4 from Iran, 2 from the United Kingdom, 4 from the US, 2 from Germany, 1 from Korea, 2 from Italy, and 1 from Switzerland.), there were 2 multi-arm studies (i.e., multiple interventions), among which one was a four-arm study, one was a three-arm study, and the rest were all two-arm studies. Since intervention measures in the multi-arm study included non-physical exercise methods, a total of 30 studies were included after excluding non-physical exercise intervention methods. The subjects of the study were people with autism spectrum disorders, and the interventions included 6 types of dance sports, ball sports, equestrian sports, martial arts, water sports and comprehensive physical exercise, while the control group did not exercise regularly (Table 1).

**Table 1:** Basic features of literature included in the study.

Literature included	Countries	Sample E/C	Age (years)		Intervention type		Intervention dose			Outcome index
			Experimental group	Control group	Experimental group	Control group	Period Week	Frequency Times/week	Time Min/time	
Chan et al. (2013) (13)	China	20/20	11.28 ± 3.90	12.42 ± 3.25	NYG + Muscle relaxation	Muscle relaxation	4	2	60	ATEC
Movahedi et al. (2013) (14)	Iran	13/13	9.54 ± 3.43	9.06 ± 3.33	KD	Daily activities	14	NR	90	GARS-2
Haghighi et al. (2022) (15)	Iran	8/8	9.00 ± 1.31	8.13 ± 1.36	CPT	No intervention	8	NR	60 ~ 70	GARS-2
Harris et al. (2017) (16)	Britain	10/14	7.96 ± 0.78	6.97 ± 0.33	THR	No intervention	7	NR	45	CARS-2/ABC-C
Tse (2020) (17)	China	15/12	10.07 ± 1.10	9.42 ± 0.90	CPT	Daily activities	12	4	30	CBCL
Coman et al. (2017) (18)	America	25/25	8.70 ± 1.60	8.70 ± 1.60	THR	Daily activities	12	1	70	SRS
Nekar et al. (2022) (19)	South Korea	12/12	14.42 ± 5.14	14.17 ± 5.09	CPT + Cognitive therapy	Cognitive therapy	4	2	30	RRBs
Bahrami et al. (2015) (20)	Iran	15/15	9.20 ± 3.32	9.06 ± 3.33	KD + Educational intervention	Educational intervention	14	4	90	GARS-2
Caputo et al. (2018) (21)	Italy	13/13	8.3 ± 2.30	7.7 ± 2.00	AT + Conventional treatment	Conventional treatment	24	1	45	CARS/VABS
Moradi et al. (2020) (22)	Iran	25/25	1.03 ± 7.64	1.25 ± 7.20	SIT	Daily activities	8	2	50	GARS-2
Marzouki et al. (2022) (23)	Switzerland	16/12	6.3 ± 0.50	6.3 ± 0.50	AT	Daily activities	8	2	50	GARS-2
Phung et al. (2021) (24)	America	14/20	9.10 ± 1.10	9.52 ± 1.07	MMAT	Daily activities	13	2	45	SSIS
Wang et al. (2020) (25)	China	18/15	5.11 ± 0.65	4.70 ± 0.70	MBTP	Daily activities	12	5	40	SRS-2/RBS-R
Cai et al. (2020) (26)	China	15/14	5.13 ± 0.61	4.68 ± 0.72	MBTP	Daily activities	12	5	40	SRS-2
Cai et al. (2020) (27)	China	30/29	4.56 ± 0.84	5.03 ± 0.64	MBTP	Daily activities	12	5	40	SRS-2
Hildebrandt et al. (2016) (28)	Germany	53/22	23.07 ± 8.54	21.27 ± 5.32	DMT	No intervention	10	NR	60	SANS
Bass et al. (2009) (29)	America	19/15	6.95 ± 1.67	7.73 ± 1.65	THR + Conventional treatment	Conventional treatment	12	1	60	SRS
Zanobini et al. (2019) (30)	Italy	13/12	5.69 ± 1.27	5.42 ± 1.54	AT + Conventional treatment	Conventional treatment	24	0.5	30	SRS/ABC
Gabriels et al. (2015) (31)	America	58/58	10.5 ± 3.20	10.0 ± 2.70	THR	Daily activities	10	NR	45	SRS/VABS-II
Koch et al. (2015) (32)	Germany	16/15	22.00 ± 7.70	22.00 ± 7.70	DMT	No intervention	7	NR	60	FBT
Yang et al. (2021) (33)	China	15/15	5.03 ± 0.55	4.67 ± 0.70	MBTP+ Routine rehabilitation	Routine rehabilitation	12	5	40	SRS-2
Aithal et al. (2021) (34)	Britain	10/16	11.53	9.77	DMP + Routine nursing	Routine nursing	5	2	40	SCQ
Xu et al. (2019) (35)	China	50/53	6.17 ± 2.44	6.18 ± 2.94	SIT + Conventional treatment	Conventional treatment	16	NR	NR	CARS
Dong et al. (2020) (36)	China	15/15	4.67 ± 0.70	4.97 ± 0.61	MBTP + Routine rehabilitation	Routine rehabilitation	12	5	40	RRBs
Liu et al. (2021) (37)	China	13/10	8.23 ± 1.30	8.10 ± 1.37	CPT	Daily activities	6	4	60	SRS
Song et al. (2020) (38)	China	46/46	8.27 ± 0.68	8.39 ± 0.53	AT + Routine nursing	Routine nursing	16	2 ~ 3	90	ATEC
Wang et al. (2020) (39)	China	28/26	7.36 ± 1.65	5.6. ± 1.62	AT + Routine nursing	Routine nursing	8	NR	90	ABC
Xiong et al. (2021) (40)	China	50/50	7.75 ± 1.08	8.03 ± 1.97	CPT + Conventional treatment	Conventional treatment	10	2	60	ABC
Yang et al. (2016) (41)	China	40/40	4.90 ± 1.26	4.90 ± 1.26	CPT + Conventional education	Conventional education	24	6	90	CARS
Zhang et al. (2017) (42)	China	30/30	7.62 ± 3.14	7.54 ± 2.96	CPT + Conventional treatment	Conventional treatment	24	7	NR	CARS

THR, therapeutic riding; MBTP, mini basketball training; DMT, DMP, dance movement training; CPT, comprehensive physical training; AT, aquatic training; SIT, sensory integration training; NYG, Chinese Traditional Mind–body Movement Inner Yang Gong in the Zen form; MMAT, mixed martial arts training; KD, karate training; NR, not reported; SRS-2, Social Response Scale 2nd Edition; RBS-R, Repetitive Behavior Scale Revision; FBT, Bipolar Exercise Therapy Questionnaire; SCQ, Social Interaction Questionnaire; VABS, Adaptive Behavior Scale; VABS-II, Adaptive Behavior Scale 2nd Edition; SANS, Negative Symptom Assessment Scale, SSIS, Social Skills Improvement System; CBCL, Children’s Emotional and Behavioral Functioning; ABC, Autism Behavior Checklist; SRS-2, Social Response Scale 2nd Edition; GARS-2, Autism Rating Scale; CARS, Autism Rating Scale.

### 3.3 Quality evaluation of the literature included

**Figure 2:**
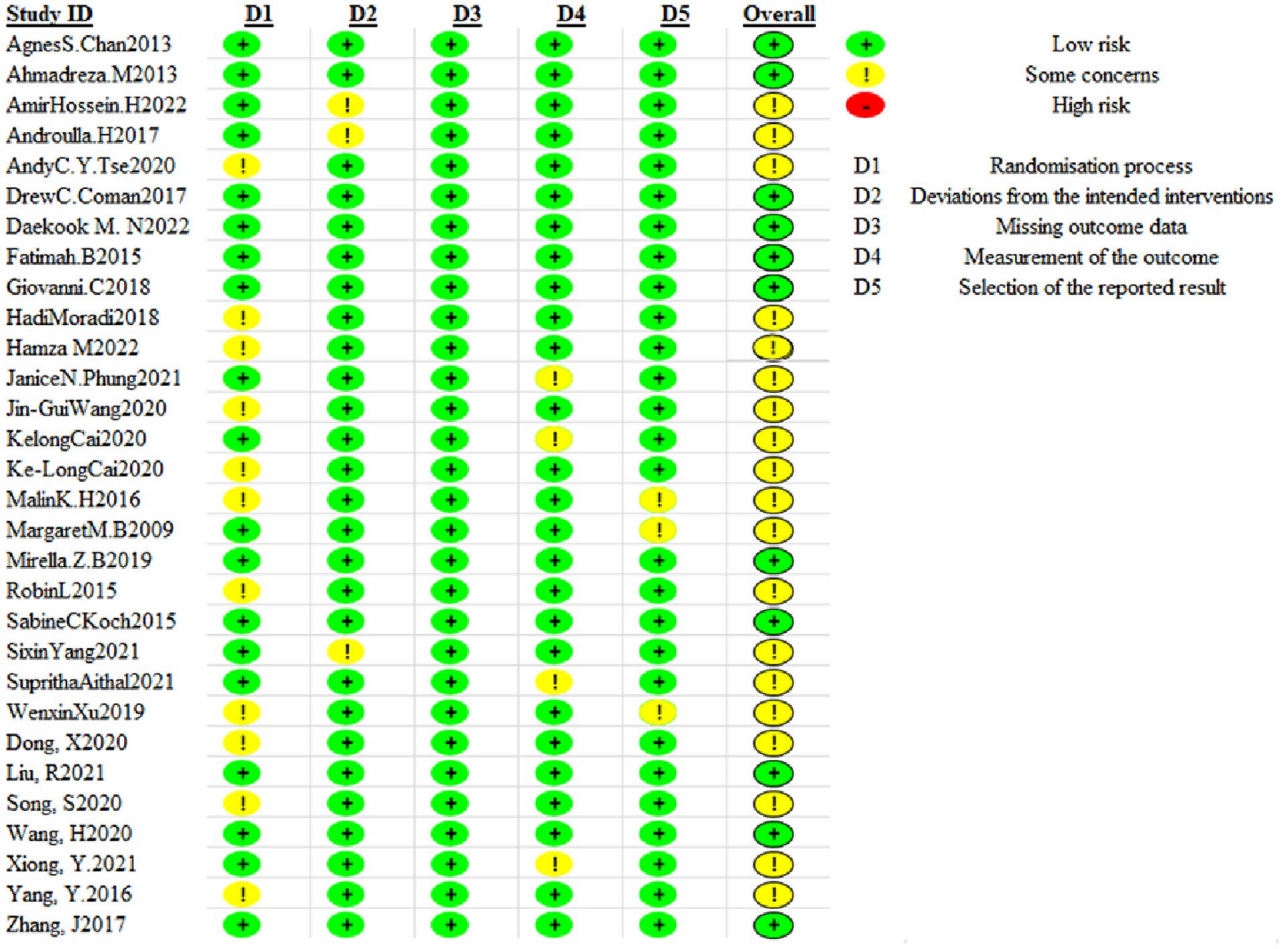
Assessment of methodological quality included in the study.

The 30 included papers were all randomized controlled trials and the results showed that: 19 studies specified the process of randomization and were at low risk of bias; 27 studies described the intended intervention and were at low risk of bias; 30 studies had no bias due to missing outcome data; 26 studies had no bias in the measurement of outcome; and 27 studies had no bias in the choice of outcome reporting. The final quality rating of the included literature was A for 11 documents, B for 19 documents and C for 0 documents (Figure 2). Two independent reviewers (SJ and XW) each assessed the quality of the literature during the literature quality assessment process, and the Cohen’s kappa value for both researchers was 0.81, indicating good agreement.

### 3.4 The combined effect size of physical exercise to improve core symptoms

The combined effect size of the 27 studies included with social interaction disorders was SMD = −0.43, 95%CI: −0.62, −0.24, suggesting that physical exercise had a significant improvement effect. Heterogeneity test results showed that there was moderate heterogeneity in studies on the effects of physical exercise on social interaction disorders in patients with autism spectrum disorder (*I*^2^ = 59%, *p* < 0.001; Table 2).

**Table 2:** Combined effect size and adjustment effect results.

Moderator variable	*I* ^2^	*n* (ES)	SMD, 95%CI	*p* 值
Social interaction disorder	59	27	−0.43 [−0.62, −0.24]	<0.001
Exercise types	68.1	6		<0.050^a^
Dance sports	23	3	0.18 [−0.29, 0.66]	0.450
Ball sports	0	4	−0.92 [−1.29, −0.54]	<0.001
Equestrian sports	0	4	−0.38 [−0.65, −0.11]	<0.050
Martial arts	74	4	−0.24 [−0.96, 0.47]	0.500
Water sports	74	4	−0.24 [−0.84, 0.35]	0.420
Comprehensive physical exercise	43	8	−0.64 [−0.91, −0.38]	<0.001
Intervention time (min/time)	54.1	3		0.110^a^
≤45	61	11	−0.40 [−0.60, −0.20]	<0.001
45 ~ 60	77	7	−0.32 [−0.55, −0.09]	<0.050
>60	0	7	−0.64 [−0.85, −0.42]	<0.001
Intervention cycle (weeks)	74.6	3		<0.050^a^
≤8	0	8	−0.12 [−0.36, 0.12]	0.330
8 ~ 12	56	10	−0.66 [−0.95, −0.36]	<0.001
>12	68	9	−0.43 [−0.78, −0.09]	<0.050
Frequency of intervention (times/week)	81.5	3		<0.050^a^
≤3	73	13	−0.13 [−0.47, 0.21]	0.470
3 ~ 5	0	8	−0.84 [−1.11, −0.57]	<0.001
>5	0	2	−0.69 [−1.03, −0.35]	<0.001
Ages of patients (years)	71.5	3		<0.05^a^
3 ~ 6	0	5	−0.81 [−1.10, −0.52]	<0.001
6 ~ 12	62	15	−0.39 [−0.65, −0.14]	<0.050
>12	59	7	−0.22 [−0.60, 0.17]	0.270
Repetition of the stereotyped behavior	61	21	−0.53 [−0.76, −0.29]	<0.001
Exercise types	39.8	6		0.140^a^
Dance sports			−0.24 [−1.03, 0.56]	0.560
Ball sports	0	5	−0.70 [−1.04, −0.36]	<0.001
Equestrian sports	76	4	−0.18 [−0.45, 0.09]	0.190
Martial arts	70	2	−0.46 [−0.93, 0.02]	0.060
Water sports	0	3	−0.15 [−0.69, 0.40]	0.600
Comprehensive physical exercise	0	7	−0.56 [−0.80, −0.33]	<0.001
Intervention time (min/time)	53.7	3		0.120^a^
≤45	16	12	−0.43 [−0.63, −0.24]	<0.001
45 ~ 60	82	5	−0.43 [−0.77, −0.09]	<0.05
>60	0	3	−0.83 [−1.17, −0.49]	<0.001
Intervention cycle (weeks)	40.6	3		0.190^a^
≤8	67	8	−0.36 [−0.63, −0.09]	<0.050
8 ~ 12	52	9	−0.37 [−0.58, −0.15]	<0.001
>12	25	4	−0.68 [−0.97, −0.39]	<0.001
Frequency of intervention (times/week)	73.2	3		0.020^a^
≤3	47	10	−0.26 [−0.58, 0.07]	0.120
3 ~ 5	60	7	−0.98 [−1.45, −0.50]	<0.001
>5	0	2	−0.75 [−1.09, −0.41]	<0.001
Ages of patients (years)	69.2	3		0.040^a^
3 ~ 6	0	7	−0.64 [−0.90, −0.38]	<0.001
6 ~ 12	72	11	−0.59 [−1.01, −0.17]	<0.050
>12	0	3	−0.15 [−0.45, 0.14]	0.310

The age of the subgroup analysis was divided according to Erikson’s eight-stage theory: Early school age (3–6 years old), School age (6–12 years old), Adolescence and above (12-years old). “^a^”: The *p*-value of the regulating factor.

The combined effect size of the repetition of the stereotyped behavior included in 22 studies was SMD = −0.53, 95%CI: −0.76, −0.29, suggesting that physical exercise had a significant improvement effect. Heterogeneity results showed that there was moderate heterogeneity in studies on the effect of physical exercise on the repetition of the stereotyped behavior in patients with autism spectrum disorders (*I*^2^ = 61%, *p* < 0.001; Table 2).

### 3.5 Moderating effects of different subgroups on outcome indicators

Subgroup analysis showed that in terms of exercise types, ball games and comprehensive physical exercise had significant improvement effect on social interaction disorders and the repetition of the stereotyped behavior (*p* < 0.05). What’s more, equestrian sports only had significant improvement effect on social interaction disorders (*p* < 0.05). Finally, dancing, martial arts and water sports had no improvement effect on social interaction disorders and the repetition of the stereotyped behavior (*p* > 0.05). In terms of the intervention time: exercise in 45 min and less, 45 ~ 60 min and more than 60 min could improve social interaction disorders and the repetition of the stereotyped behavior significantly (*p* < 0.05). In terms of intervention cycle: the physical exercise of 8 weeks or less had no improvement effect on social interaction disorder (*p* > 0.05) and the physical exercise of 8 weeks had significant improvement effect on social interaction disorder as well as the repetition of the stereotyped behavior (*p* < 0.05). In terms of the frequency of intervention: physical exercise of 3 times/week or less had the significant effect on the improvement of social interaction disorder and the repetition of the stereotyped behavior (*p* < 0.05) and physical exercise of more than 3 times a week had a significant effect on the improvement of social interaction disorder as well as the repetition of the stereotyped behavior (*p* < 0.05). In terms of patient age, the improvement effect was significant in patients aged 12 years and below (*p* < 0.05), while the improvement effect was not significant in patients aged over 12 years (*p* > 0.05; Table 2).

### 3.6 The evidence network of movement element combination

**Figure 3:**
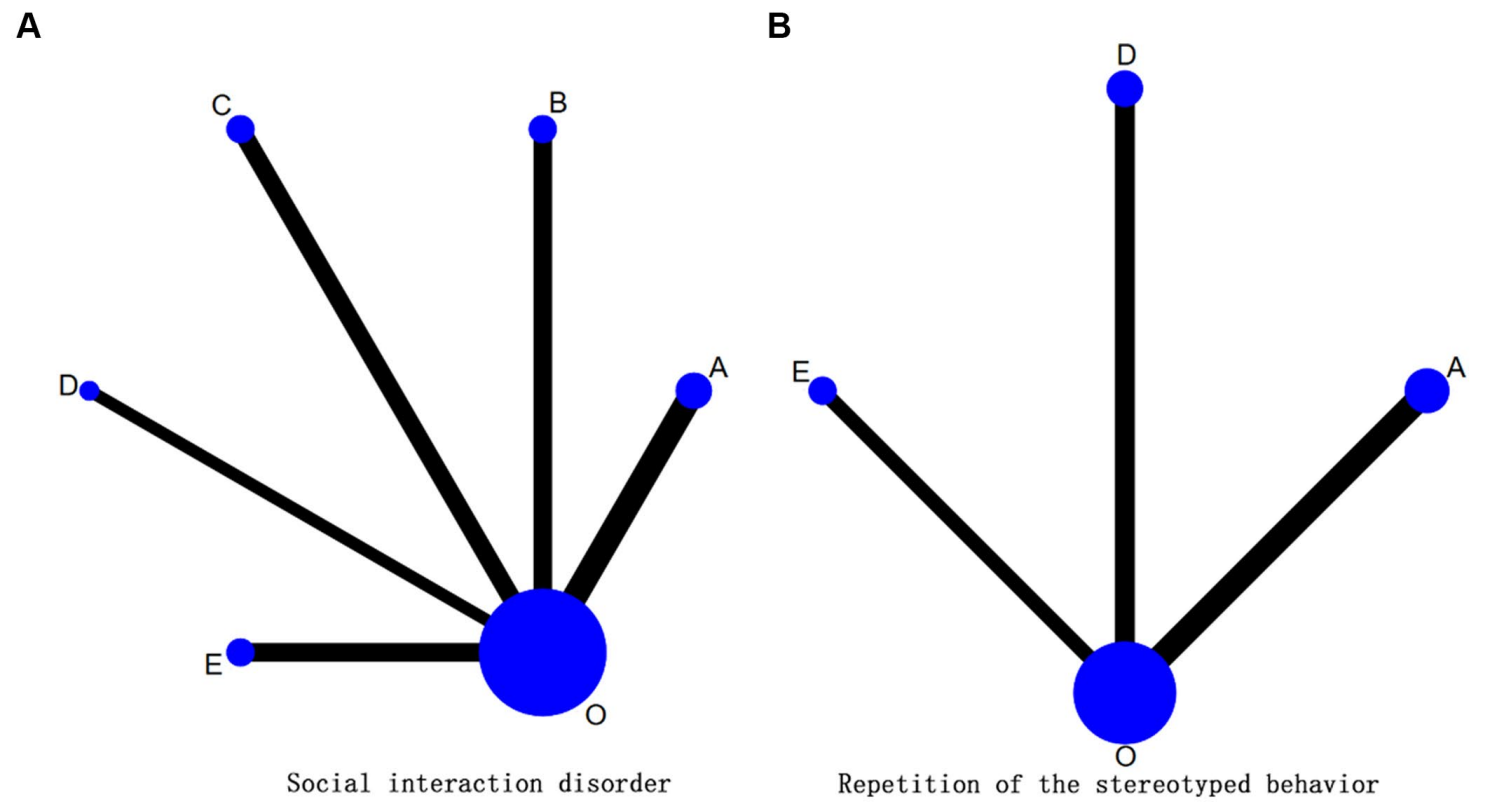
**(A, B)** The evidence network diagram of physical exercise intervention in core symptoms of patients with autism spectrum disorders. “0” Represented the control group (routine interventions, daily activities, educational intervention and no intervention). A: ball games for 8–12 weeks; D: comprehensive physical exercise for 8–12 weeks; E: comprehensive physical exercise for 12 weeks, O: control group (no exercise intervention).

In this paper, the combination of exercise elements was discussed, and the subgroup analysis found that there existed significant differences in exercise type, intervention period and frequency. The heterogeneity of intervention time was low and no difference existed. Since intervention frequency was not reported in some studies, so based on traditional meta-analysis, mesh meta-analysis was conducted on exercise type and intervention period (Figure 3). A total of 27 studies (1,219 cases) were included in the evidence network. The overall consistency test showed that the overall difference between the consistent model and the inconsistent model of the social interaction disorder (DIC = 47.12/ DIC = 47.28) and the repetition of the stereotyped behavior (DIC = 40.09/ DIC = 40.12) was less than 1, indicating there existed a good overall consistency, so the consistency model was used for analysis. A total of 15 studies (787 cases) related to social interaction disorders were included, involving five exercise regimen (Figure 3) in which PSRF converged to 1, and the overall heterogeneity was low (*I*^2^ = 0%). A total of 12 studies (432 cases) related to the repetition of the stereotyped behavior were included, involving three exercise regimens (Figure 3) in which PSRF converged to 1, and the overall heterogeneity was low (*I*^2^ = 0%).

### 3.7 Optimization of physical exercise to improve core symptoms

As to the outcome indicators of social interaction disorder, 8–12 weeks of ball sports, 8–12 weeks of equestrian sports, and more than 8 weeks of comprehensive physical exercise could significantly improve the social interaction disorder of patients with autism spectrum disorder (*p* < 0.05), but the effect of comprehensive physical exercise intervention after 8 weeks was not significant. According to the SUCRA value, the effects of different interventions on the improvement of social interaction disorders were ranked. Table 3 showed that the intervention effects of the five exercise methods were ranked from high to low as follows: ball games for 8–12 weeks > comprehensive physical exercise for more than 12 weeks > equestrian sports for 8–12 weeks > comprehensive physical exercise for 8–12 weeks > comprehensive physical exercise for 8 weeks and less than 8 weeks. Indirect comparison of different exercise programs showed that ball games in 8–12 weeks had significant differences with comprehensive physical exercise for 8 weeks or less as well as comprehensive physical exercise for 8–12 weeks, but had no significant differences with equestrian sports for 8 to 12 weeks and comprehensive physical exercise for more than 12 weeks. What’s more, there existed significant difference between equestrian sports for 8–12 weeks and comprehensive physical training for more than 12 weeks, but there was no significant difference between equestrian sports and other forms of exercise. There was no significant difference between comprehensive physical exercise for 8 weeks or less and more than 8 weeks, and there was no significant difference between comprehensive physical exercise for 8–12 weeks and more than 12 weeks.

**Table 3:** Intervention effect and SUCRA value of social interaction disorder.

	A	B	C	D	E	O	Socialization (SUCRA)	Behavior (SUCRA)
A				3.15 (−0.63 6.09)	0.79 (−2.61, 3.97)	4.36 (2.04, 6.73)*	0.93	0.88
B	−1.66 (−5.07, 1.60)						0.67	
C	−4.01 (−7.95, −0.12)*	−2.35 (−6.45, 1.80)					0.28	
D	−3.17 (−5.98, −0.42)*	−1.54 (4.52, 1.65)*	0.813 (−2.85, 4.55)		−2.39 (−5.26, 1.34)	1.14 (−0.64, 4.20)	0.39	0.34
E	−1.59 (−4.96, 1.73)	0.03 (−3.44, 3.76)	2.40 (−1.65, 6.55)	1.58 (−1.46, 4.67)		3.56 (1.40, 6.08)*	0.68	0.74
O	−5.35 (−7.57, −3.23)*	−3.71 (−6.18, −1.13)*	−1.36 (−4.60, 1.91)	−2.17 (−3.99, −0.44)*	−3.75 (−6.33, −1.24)*		0.05	0.03

**Figure 4:**
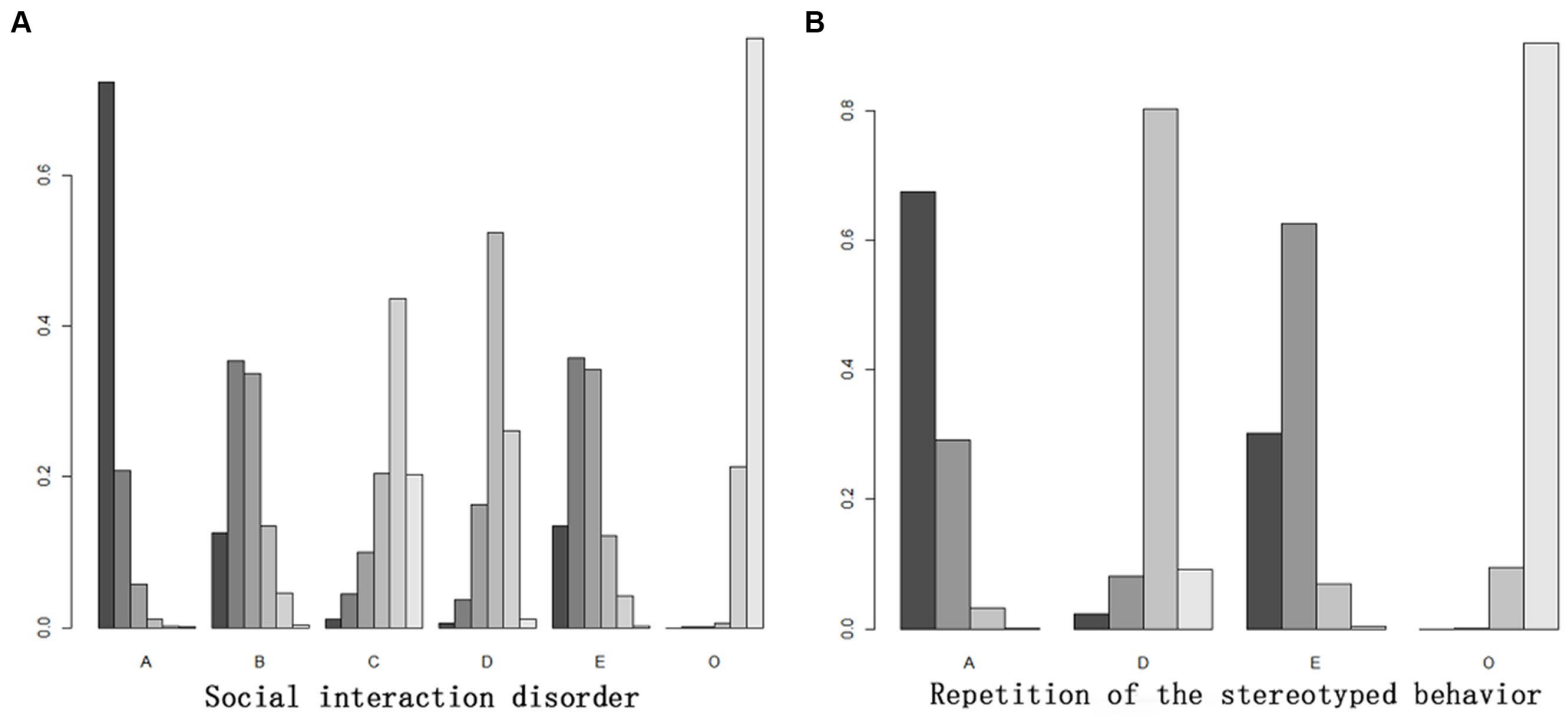
**(A, B)** Probability ranking diagram on intervention effect of each outcome index. “O” represented the control group (routine intervention, daily activities, educational intervention and no intervention). A ball: games for 8–12 weeks; B: equestrain exercise for 8–12 weeks; C: comprehensive physical activities for 8–12 weeks; O: control group (no exercise intervention).

The lower left part of the table was the pair comparison results of social interaction disorders, and the upper right part was the pair comparison results of the repetition of the stereotyped behavior. “*”: Indicates statistically significant difference (*p* < 0.05). The grouping in Table 3 is shown in Figure 4.

As for the outcome index of the repetition of the stereotyped behavior, ball games for 8–12 weeks and comprehensive physical exercise for more than 12 weeks could significantly improve the repetition of the stereotyped behavior in patients with autism spectrum disorder (*p* < 0.05), while comprehensive physical exercise for 8–12 weeks had no significant effect. According to SUCRA value, the effects of different interventions on improving the repetition of the stereotyped behavior were ranked. Table 3 showed that the intervention effects of the three types of exercise were ranked from high to low as: ball games for 8–12 weeks > comprehensive physical exercise for more than 12 weeks > comprehensive physical exercise for 8–12 weeks. Indirect comparison of different exercise regimens found that there was no significant difference in indirect comparison between exercise regimens and the control group (Table 3; Figure 4).

### 3.8 Sensitivity analysis

To investigate whether the heterogeneity between studies was caused by individual studies, this review performed sensitivity analyses on the core symptom outcome indicators and analysed the combined effect by screening out individual studies one at a time. The sensitivity analysis of the included study outcome indicators is reported in Appendix 10 of the eAddenda. The combined effect size of social interaction disorders included in all studies was SMD = −0.43, 95% CI (−0.62, −0.24), *p* < 0.001, *I*^2^ = 59%, and the range of combined effect SMDs after screening out individual studies was (−0.48 to −0.40), and the range of I^2^ was (56.86–64.14%), all with *p* < 0.001. Repeated stereotypic behavior included in all studies had a combined effect size SMD = −0.53, 95% CI (−0.76, −0.29), *p* < 0.001, *I*^2^ = 61%, the range of the combined effect SMD after sifting out the individual studies was (−0.60 to −0.46) and the range of *I*^2^ was (44.37–66.30%), all with P less than 0.001, with Liu, R2021 this study causing larger *I*^2^ changes. The results of the two analyses showed that the relatively low sensitivity of the data in this study did not fundamentally change the results of the meta-analysis, suggesting that the results of the study’s outcome metrics are somewhat stable and reliable.

### 3.9 Publication bias

**Figure 5:**
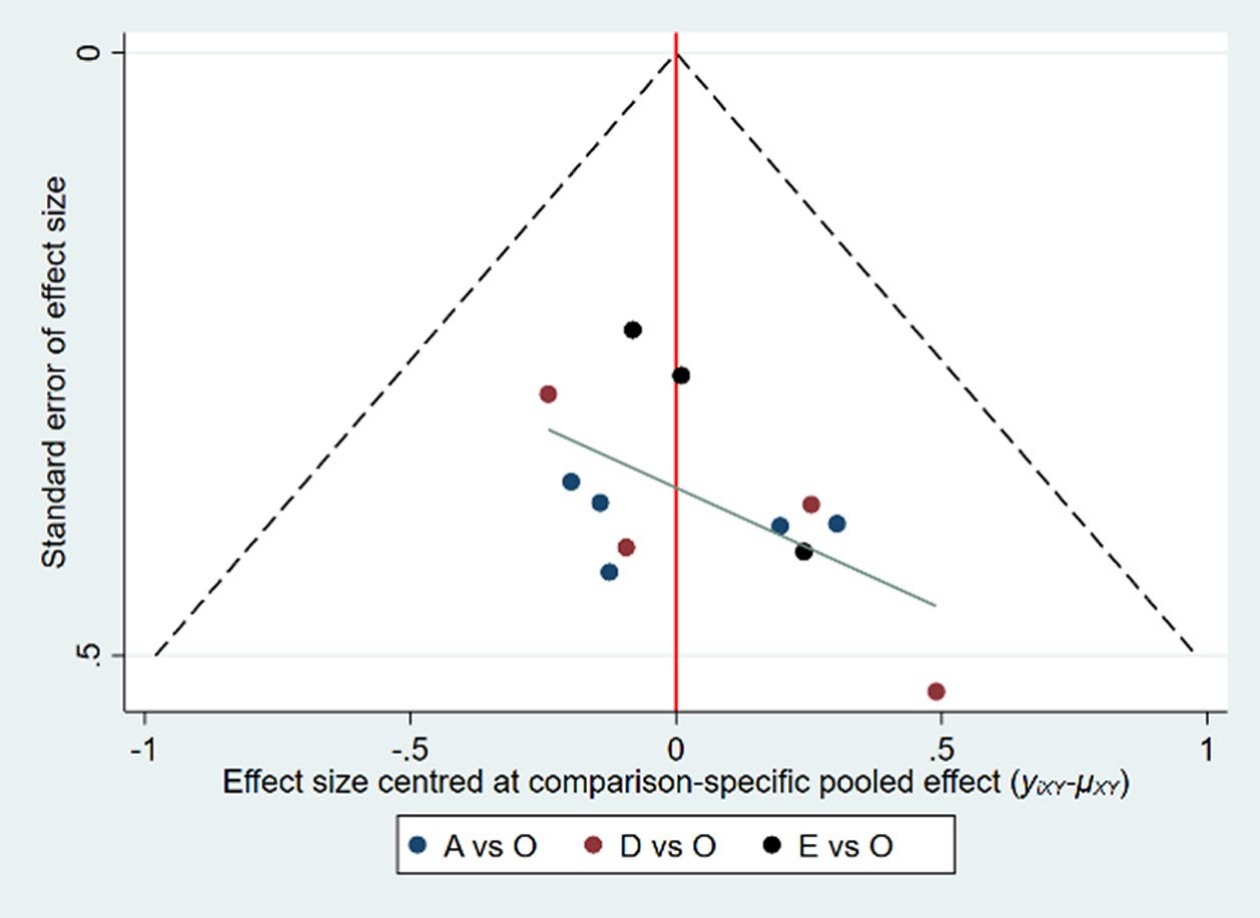
The inverted funnel diagram of exercise improving the repetition of the stereotyped behavior in patients with autism spectrum disorder.

The inverted funnel analysis was conducted using the repetition of the stereotyped behavior in patients with autism spectrum disorders as outcome indicators. As shown in Figure 5, the funnel plot presented asymmetry, and some black dots were still at the lower part of the funnel plot, suggesting that the repetition of the stereotyped behavior as outcome indicators in this study might have certain small sample effect and publication bias, and the results should be treated with caution. The outcome index of social interaction disorder basically maintained symmetry, and there was no obvious deviation.

### 3.10 Certainty of the network meta-analysis evidence

The overall certainty of the evidence available for each comparison in the primary network meta-analyses, assessed using the CINeMA framework, is reported in Appendix 10 on the eAddenda. The findings demonstrate that the direct comparisons concerning the social interaction disorder and repetitive stereotyped behaviors outcome indicators had a predominantly moderate quality of evidence. In contrast, the evidence quality for indirect comparisons was lower because of the lack of loop closure and the high inconsistency levels observed.

## 4 Discussion

This study showed that physical exercise had positive effects on social interaction disorders and the repetition of the stereotyped behavior in patients with autism spectrum disorder, which was consistent with previous results (43, 44). ASD patients participated in sports activities as a group, learned social etiquette (27), induced and strengthened social communication, improved eye processing ability and attention (45, 46), and finally improved cognitive neural function (47, 48), which had a positive impact on brain function activation (36, 49), and improved social barriers. In addition, physical exercise was similar to the stereotyped behavior of ASD patients. The target activities of individuals replaced the non-target stereotyped activities, enhancing their internal needs, and patients would no longer need to experience pleasure from stereotyped behaviors (22).

The frequency and cycle of physical exercise were important factors that could affect the effect of intervention. We found that physical exercise for more than three times a week was an effective tool to reduce the number of stereotypical behavior episodes in children with ASD (50). With the extension of intervention period, the overall intervention effect of physical exercise might be better. There was evidence that physical exercise for 5 times a week could reduce functional magnetic resonance imaging (fMRI) brain activation in the prefrontal cortex, which might lead to more mature brain function (51). Long-term participation in physical activity could lead to changes in the expression of genes related to the synthesis and release of neurotransmitters (44), and induce the adaptation of brain structure and synaptic plasticity (52), thus affecting the enhancement of neural function and improve cognitive and behavioral abilities. The intervention effects of different exercise forms were different from previous studies. Martial arts, swimming and dancing could not produce significant effects on patients with ASD (43, 53). In addition, we found that the age of physical exercise intervention was an important factor affecting the effect size, and the effect of intervention was more significant in patients under 12 years of age. Early diagnosis and intervention could reduce complications and related disabilities in patients with ASD (54), which could better improve the educational performance and cognitive development of children with ASD (3), and help them better adapt to daily life (55) by changing functional prediction and timely referral to specialists who assess and treat early symptoms.

By combining the results of direct comparison and indirect comparison, the pair-to-pair comparison and quantitative ranking of various interventions showed that the exercise programs that could significantly improve social interaction disorders were ball games for 8 to 12 weeks, comprehensive physical exercise for more than 12 weeks, equestrian sports for 8–12 weeks and comprehensive physical exercise for 8–12 weeks. The exercise programs that could significantly improve the repetition of the stereotyped behavior were ball games for 8–12 weeks and comprehensive physical exercise for more than 12 weeks. Equestrian exercise and comprehensive physical exercise for 8–12 weeks can only improve the social interaction disorders of ASD patients, suggesting that the exercise program had a selective effect on outcome indicators.

This study found that the intervention effects of different forms of exercise differed from previous studies. Firstly, martial arts, swimming and dance interventions did not produce improvement in patients with ASD, and this discrepancy may be related to the low intervention frequency of the exercise programmes included in the study. The intervention frequency of dance exercise, swimming exercise and martial arts exercise in the studies included in this paper was less than 3 times/week, and physical activity less than 3 times/week did not have an ameliorative effect on symptoms of ASD, which is consistent with the results shown in the subgroup analysis. Second, the same exercise programme may have selective effects on different outcome indicators, and the improvement effects of combined physical activity did not occur simultaneously, with equine exercise only improving social interaction deficits in ASD patients. Physical activity has transient and chronic beneficial effects on repetitive stereotypic behaviors in people with ASD, but these effects are usually facilitated by a single exercise modality, and combinations of exercise modalities allow more possibilities for intervention mechanisms, but studies have found that the effects of combinations of multiple exercise modalities may take longer to activate and change, which may be a possible reason for the cyclical selection of combined physical activity when intervening in repetitive stereotypic behaviors. The presence of an important active ingredient in human-horse interaction maintains a relaxing environment that can influence positive changes in irritability, hyperactivity, and social and communicative behaviors in this population (18), which may have a calming effect on children with autism, which in turn improves their social interactions (29); however, the lack of training in equestrian sports may have influenced the effect of improving repetitive stereotypic behaviors in the patient’s behavior. However, The intervention effect of ball sports on social interaction disorders and repetitive and rigid behaviors occurs simultaneously. In the form of collective classroom, ball sports can induce and strengthen social communication, improve cognitive nerves (53), exert a positive impact on brain function activation (44), and promote the enhancement of brain plasticity by watching peer exercises and imitating exercises to learn social etiquette. Thus improving the social ability of children with ASD. In addition, through the learning of basic motor skills, ball sports can produce a pleasant state similar to stereotypical behaviors (56), and at the same time increase the gray matter volume of the right cerebellar area 8 of preschool children with ASD (57), improve their sensory and perceptual abilities and behavioral control, so that the repetitive stereotypical behaviors of children with ASD can be controlled. Similar findings were found in karate training (20).

To sum up, early intervention was crucial for patients with autism spectrum disorders. Evidence showed that 8–12 weeks of ball exercise for 5 times a week had the best performance in improving core symptoms. Subgroup analysis suggested that long-term intervention was more effective.

Limitations and implications of this study: (1) Exercise intensity was an important factor in intervention programs. Due to the particularity of patients with autism spectrum disorders, it was difficult to use instruments to measure exercise intensity, which might result in the heterogeneity; (2) Most outcome indicators included in this study were judged by scales, which were easily affected by subjective factors. Therefore, more objective evaluation tools should be introduced in the future. (3) The incompleteness of the feedback loop in the network analyses of the comparisons of various exercise interventions could have impeded the outcomes of both direct and indirect comparisons.

## Data availability statement

The original contributions presented in the study are included in the article/Supplementary material, further inquiries can be directed to the corresponding author.

## Author contributions

LL: Writing – review & editing, Writing – original draft, Validation, Conceptualization. SJ: Writing – review & editing, Writing – original draft, Investigation, Data curation. PW: Writing – review & editing, Formal analysis. SL: Writing – review & editing, Investigation. XW: Writing – review & editing, Validation, Supervision. XZ: Writing – review & editing.
