# Supplementary material for: A network meta-analysis of the effect of physical exercise on core symptoms in patients with autism spectrum disorders
Source: Front Neurol. 2024 May 9;15:1360434. doi: 10.3389/fneur.2024.1360434 (PMC11113547; doi:10.3389/fneur.2024.1360434)
Supplement: Supplementary file 1 [file Data_Sheet_1.docx]

**A network meta-analysis of the effect of physical exercise on core symptoms in patients with autism spectrum disorders**

| SUPPLEMENTAL MATERIALS | Page |
| --- | --- |
| Appendix 1. Completed PRISMA-NMA checklist. | 2-5 |
| Appendix 2. The complete record of electronic databases that underwent search and the "Physical Exercise for Autism Core Symptoms RCT Trial" exemplify a Cochrane search procedure. | 5-6 |
| Appendix 3. Exercise characteristics definitions for included trials. | 6 |
| Appendix 4. Analysis code and additional information. | 7-8 |
| Appendix 5. Summary table of pairwise comparison results including direct data available, direct comparison meta-analysis summary estimates, and mixed NMA estimates. | 7-8 |
| Appendix 6. Description of CINeMA domain assessments: risk of bias, indirectness, incoherence, heterogeneity, imprecision, publication bias. | 6-10 |
| Appendix 7. Citations for 53 included trials. | 11-12 |
| Appendix 8. Detailed table of included trials. Description of each included trial including population, setting, exercise and comparison treatments | 12-14 |
| Appendix 9. Detailed risk of bias assessments for each included trial. | 15 |
| Appendix 10. Sensitivity analysis of included study outcome indicators | 15-16 |
| Appendix 11. Judgments for each domain and overall certainty rating for each pairwise comparison (CINeMA assessment). | 16 |

Appendix 1. PRISMA NMA Checklist of Items to Include When Reporting A Systematic Review Involving a Network Meta-analysis

Study: A Networkmeta-analysis of the effect of physical exercise on core symptoms in patients with autism spectrum disorders

| **Section/Topic** | **Item #** | **Checklist Item** | **Reported on Page #** |
| --- | --- | --- | --- |
| **TITLE** |  |  |  |
| Title | 1 | Identify the report as a systematic review *incorporating a network meta-analysis (or related form of meta-analysis).* |  |
| **ABSTRACT** |  |  |  |
| Structured summary | 2 | Provide a structured summary including, as applicable:  **Background:** main objectives  **Methods:** data sources; study eligibility criteria, participants, and interventions; study appraisal; and *synthesis methods, such as network meta-analysis.*  **Results:** number of studies and participants identified; summary estimates with corresponding confidence/credible intervals; *treatment rankings may also be discussed. Authors may choose to summarize pairwise comparisons against a chosen treatment included in their analyses for brevity.*  **Discussion/Conclusions:** limitations; conclusions and implications of findings.  **Other:** primary source of funding; systematic review registration number with registry name. |  |
| **INTRODUCTION** |  |  |  |
| Rationale | 3 | Describe the rationale for the review in the context of what is already known*, including mention of why a network meta-analysis has been conducted.* |  |
| Objectives | 4 | Provide an explicit statement of questions being addressed, with reference to participants, interventions, comparisons, outcomes, and study design (PICOS). |  |
| **METHODS** |  |  |  |
| Protocol and registration | 5 | Indicate whether a review protocol exists and if and where it can be accessed (e.g., Web address); and, if available, provide registration information, including registration number. |  |
| Eligibility criteria | 6 | Specify study characteristics (e.g., PICOS, length of follow-up) and report characteristics (e.g., years considered, language, publication status) used as criteria for eligibility, giving rationale. *Clearly describe eligible treatments included in the treatment network, and note whether any have been clustered or merged into the same node (with justification).* |  |
| Information sources | 7 | Describe all information sources (e.g., databases with dates of coverage, contact with study authors to identify additional studies) in the search and date last searched. |  |
| Search | 8 | Present full electronic search strategy for at least one database, including any limits used, such that it could be repeated. |  |
| Study selection | 9 | State the process for selecting studies (i.e., screening, eligibility, included in systematic review, and, if applicable, included in the meta-analysis). |  |
| Data collection process | 10 | Describe method of data extraction from reports (e.g., piloted forms, independently, in duplicate) and any processes for obtaining and confirming data from investigators. |  |
| Data items | 11 | List and define all variables for which data were sought (e.g., PICOS, funding sources) and any assumptions and simplifications made. |  |
| **Geometry of the network** | **S1** | Describe methods used to explore the geometry of the treatment network under study and potential biases related to it. This should include how the evidence base has been graphically summarized for presentation, and what characteristics were compiled and used to describe the evidence base to readers. |  |
| Risk of bias within individual studies | 12 | Describe methods used for assessing risk of bias of individual studies (including specification of whether this was done at the study or outcome level), and how this information is to be used in any data synthesis. |  |
| Summary measures | 13 | State the principal summary measures (e.g., risk ratio, difference in means). *Also describe the use of additional summary measures assessed, such as treatment rankings and surface under the cumulative ranking curve (SUCRA) values, as well as modified approaches used to present summary findings from meta-analyses.* |  |
| Planned methods of analysis | 14 | Describe the methods of handling data and combining results of studies for each network meta-analysis. This should include, but not be limited to:   - *Handling of multi-arm trials;* - *Selection of variance structure;* - *Selection of prior distributions in Bayesian analyses; and* - *Assessment of model fit.* |  |
| **Assessment of Inconsistency** | **S2** | Describe the statistical methods used to evaluate the agreement of direct and indirect evidence in the treatment network(s) studied. Describe efforts taken to address its presence when found. |  |
| Risk of bias across studies | 15 | Specify any assessment of risk of bias that may affect the cumulative evidence (e.g., publication bias, selective reporting within studies). |  |
| Additional analyses | 16 | Describe methods of additional analyses if done, indicating which were pre-specified. This may include, but not be limited to, the following:   - Sensitivity or subgroup analyses; - Meta-regression analyses; - *Alternative formulations of the treatment network; and* - *Use of alternative prior distributions for Bayesian analyses (if applicable).* |  |
| **RESULTS†** |  |  |  |
| Study selection | 17 | Give numbers of studies screened, assessed for eligibility, and included in the review, with reasons for exclusions at each stage, ideally with a flow diagram. |  |
| **Presentation of network structure** | **S3** | Provide a network graph of the included studies to enable visualization of the geometry of the treatment network. |  |
| **Summary of network geometry** | **S4** | Provide a brief overview of characteristics of the treatment network. This may include commentary on the abundance of trials and randomized patients for the different interventions and pairwise comparisons in the network, gaps of evidence in the treatment network, and potential biases reflected by the network structure. |  |
| Study characteristics | 18 | For each study, present characteristics for which data were extracted (e.g., study size, PICOS, follow-up period) and provide the citations. |  |
| Risk of bias within studies | 19 | Present data on risk of bias of each study and, if available, any outcome level assessment. |  |
| Results of individual studies | 20 | For all outcomes considered (benefits or harms), present, for each study: 1) simple summary data for each intervention group, and 2) effect estimates and confidence intervals. *Modified approaches may be needed to deal with information from larger networks.* |  |
| Synthesis of results | 21 | Present results of each meta-analysis done, including confidence/credible intervals. *In larger networks, authors may focus on comparisons versus a particular comparator (e.g. placebo or standard care), with full findings presented in an appendix. League tables and forest plots may be considered to summarize pairwise comparisons.* If additional summary measures were explored (such as treatment rankings), these should also be presented. |  |
| **Exploration for inconsistency** | **S5** | Describe results from investigations of inconsistency. This may include such information as measures of model fit to compare consistency and inconsistency models, *P* values from statistical tests, or summary of inconsistency estimates from different parts of the treatment network. |  |
| Risk of bias across studies | 22 | Present results of any assessment of risk of bias across studies for the evidence base being studied. |  |
| Results of additional analyses | 23 | Give results of additional analyses, if done (e.g., sensitivity or subgroup analyses, meta-regression analyses*, alternative network geometries studied, alternative choice of prior distributions for Bayesian analyses,* and so forth). |  |
| **DISCUSSION** |  |  |  |
| Summary of evidence | 24 | Summarize the main findings, including the strength of evidence for each main outcome; consider their relevance to key groups (e.g., healthcare providers, users, and policy-makers). |  |
| Limitations | 25 | Discuss limitations at study and outcome level (e.g., risk of bias), and at review level (e.g., incomplete retrieval of identified research, reporting bias). *Comment on the validity of the assumptions, such as transitivity and consistency. Comment on any concerns regarding network geometry (e.g., avoidance of certain comparisons).* |  |
| Conclusions | 26 | Provide a general interpretation of the results in the context of other evidence, and implications for future research. |  |
| **FUNDING** |  |  |  |
| Funding | 27 | Describe sources of funding for the systematic review and other support (e.g., supply of data); role of funders for the systematic review. This should also include information regarding whether funding has been received from manufacturers of treatments in the network and/or whether some of the authors are content experts with professional conflicts of interest that could affect use of treatments in the network. |  |

PICOS = population, intervention, comparators, outcomes, study design.

* Text in italics indicateS wording specific to reporting of network meta-analyses that has been added to guidance from the PRISMA statement.

† Authors may wish to plan for use of appendices to present all relevant information in full detail for items in this section.

Appendix 2. Complete list of electronic databases searched.

The complete record of electronic databases that underwent search and the "Physical Exercise for Autism Core Symptoms RCT Trial" exemplify a Cochrane search procedure.

We searched the following databases with no language restrictions:

PubMed: https://pubmed.ncbi.nlm.nih.gov/

Web of Science: https://webofscience.clarivate.cn/wos/alldb/basic-search

EBSCOhost: https://www.embase.com/search/quick

Cochrane Library: http://www-cochranelibrary-com-443.ca.ilibs.cn/

Chinese databases CNKI: https://www.cnki.net/

Wanfang databases: https://www.wanfangdata.com.cn/

VIP databases: http://www.cqvip.com/

Cochrane Library February 21, 2023

[Cochrane Central Register of Controlled Trials](https://www.cochranelibrary.com/)

Search Strategy:

| **#** | **Searches** | **Results** |
| --- | --- | --- |
| 1 | " Physical activity "[Mesh] | 38838 |
| 2 | (Movement):ti,ab,kw | 43758 |
| 3 | (Physical exercise):ti,ab,kw | 56126 |
| 4 | ("exercise "):ti,ab,kw | 139472 |
| 5 | ("sport"):ti,ab,kw | 13301 |
| 6 | ("Training "):ti,ab,kw | 147438 |
| 7 | or/1-6 | 270621 |
| 8 | " autistic disorder "[Mesh] | 1442 |
| 9 | (Autism Spectrum Disorder):ti,ab,kw | 3468 |
| 10 | (Disorder, Autistic):ti,ab,kw | 2346 |
| 11 | (Spectrum):ti,ab,kw | 13926 |
| 12 | (Early Infantile Autism):ti,ab,kw | 5 |
| 13 | (Disorders, Asperger):ti,ab,kw | 283 |
| 14 | (Syndrome, Asperger):ti,ab,kw | 240 |
| 15 | or/8-15 | 14884 |
| 16 | " randomized controlled trial "[Mesh] | 25729 |
| 17 | (randomized):ti,ab,kw | 1285896 |
| 18 | (controlled):ti,ab,kw | 1273739 |
| 19 | (trial):ti,ab,kw | 1119637 |
| 20 | (randomized controlled trial):ti,ab,kw | 828301 |
| 21 | (random):ti,ab,kw | 1209650 |
| 22 | (random allocation):ti,ab,kw | 112256 |
| 23 | (RCT):ti,ab,kw | 40376 |
| 24 | (RCTs):ti,ab,kw | 12439 |
| 25 | OR/16-24 | 1636405 |
| 26 | 7 AND 15 AND 25 | 1457 |

Appendix 3. Exercise characteristic definitions for included trials

A total of 31 studies were included in this research, encompassing 9 different sports forms such as horse riding, mini-basketball, dance, integrated physical training, hydrotherapy, sensory integration, traditional Chinese mind-body exercise Zen internal training gong, mixed martial arts and karate. By categorising sports into various types, the study identified dance sports, ball sports, equestrian sports, martial arts sports, aquatic sports, and integrated physical exercise. The study's net meta-analysis included the studies mentioned above.

Appendix 4. R4.2.1 Analysis code

| library("gemtc")  data <- read.csv("****.csv", sep=",", header=T)   network <- mtc.network(data)  plot(network)  model <-mtc.model(network, type="consistency",   n.chain=4,likelihood="normal",link="identity",linearModel="random")  results <- mtc.run(model, n.adapt = 20000, n.iter = 50000, thin = 1)  summary(results)  forest(relative.effect(results, "O"))  plot(results)   gelman.plot(results)   ranks <- rank.probability(results,preferredDirection = -1) print(ranks) plot(ranks) plot(ranks, beside = TRUE)  barplot(t(ranks), beside = TRUE,  col = c("lightblue", "mistyrose", "lightcyan","lavender", "cornsilk"),ylim = c(0, 1))  a <- relative.effect.table(results) write.csv(a, "***.csv")  modelume <-mtc.model(network, type="ume", n.chain=4,  likelihood="normal",link="identity",linearModel="random") resultsume <- mtc.run(modelume, n.adapt = 20000, n.iter = 50000, thin = 1) summary(resultsume)  resultnodesplit <-mtc.nodesplit(network) b<-summary(resultnodesplit)  print(b) plot(b)   result.anohe <- mtc.anohe(network,n.adapt = 20000, n.iter = 50000) summary.anohe <- summary(result.anohe) pdf("***.pdf") plot(summary.anohe, xlim=log(c(0.2, 5))) dev.off() summary.anohe  sucra <- function(ranks) {  apply(ranks, 1, function(p) {  a <- length(p)  sum(cumsum(p[-a]))/(a-1)  }) }  sucra(ranks) |
| --- |

Appendix 5. Summary table of pairwise comparison results including direct data available, direct comparison meta-analysis summary estimates, and mixed NMA treatment effect estimates.


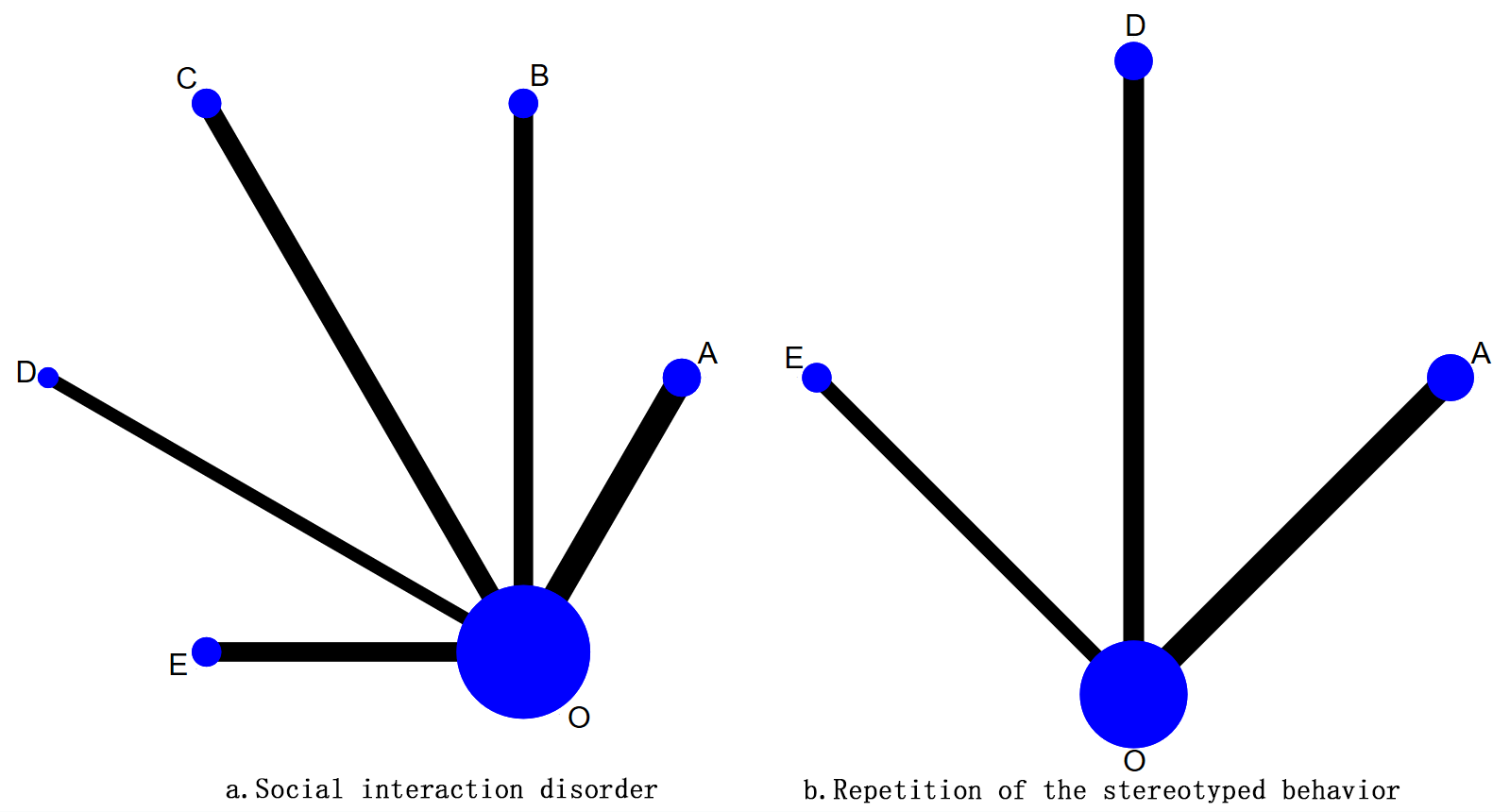


A：Social interaction disorder

|  | A | B | C | D | E | O |
| --- | --- | --- | --- | --- | --- | --- |
| A | A | 1.66(-1.60, 5.07) | 4.01(0.12, 7.95) | 3.17 (0.42, 5.98) | 1.59 (-1.73, 4.96) | 5.35 (3.23, 7.57) |
| B | -1.66 (-5.07, 1.60) | B | 2.35 (-1.80, 6.45) | 1.54 (-1.65, 4.52) | -0.03 (-3.76, 3.44) | 3.71 (1.13, 6.17) |
| C | -4.01(-7.95, -0.12) | -2.35 (-6.45, 1.80) | C | -0.81 (-4.55, 2.85) | -2.40 (-6.55, 1.65) | 1.36 (-1.91, 4.60) |
| D | -3.17 (-5.98, -0.42) | -1.54 (-4.52, 1.65) | 0.813 (-2.85, 4.55) | D | -1.58 (-4.67, 1.46) | 2.17 (0.44, 3.99) |
| E | -1.59 (-4.96, 1.73) | 0.03 (-3.44, 3.76) | 2.40 (-1.65, 6.55) | 1.58(-1.46, 4.67) | E | 3.75(1.24, 6.33) |
| O | -5.35(-7.57, -3.23) | -3.71(-6.18, -1.13) | -1.36(-4.60, 1.91) | -2.17 (-3.99, -0.44) | -3.75 (-6.33, -1.24) | O |

B：Repetition of the stereotyped behavior

|  | B | C | F | O |
| --- | --- | --- | --- | --- |
| B | B | 3.15 (-0.63 6.09) | 0.79 (-2.61, 3.97) | 4.36 (2.04, 6.73) |
| C | -3.15 (-6.09, 0.63) | C | -2.39 (-5.26, 1.34) | 1.14(-0.64, 4.20) |
| F | -0.79 (-3.97, 2.61) | 2.39 (-1.34, 5.26) | F | 3.56 (1.40, 6.08) |
| O | -4.36 (-6.73 -2.04) | -1.14(-4.20, 0.64) | -3.56 (-6.08, -1.40) | O |

Appendix 6. Description of CINeMA domain assessments: risk of bias, indirectness, incoherence, heterogeneity, imprecision, publication bias.

| CINeMA  Domain | Description of assessment |
| --- | --- |
| Risk of bias | We used the Cochrane ROB tool to assess 12 items (randomization, treatment allocation concealment, blinding of participants, care provider and outcome assessor, drop-out rate, intention to treat, selective outcome reporting, similarity at baseline, avoidance of co-interventions, compliance, and similar timing of outcome assessment) as low, unclear, or high ROB for each included study. Overall study ROB was judged to be: HIGH if ‘randomization’, ‘allocation’ or ‘drop-out’ is rated high or unclear; MODERATE if ‘randomization’, ‘allocation’ and ‘drop-out’ were ‘low’ AND any of ‘blinding’, ‘ITT’, ‘reporting’, ‘baseline’, ‘cointervention’, ‘compliance’, ‘outcome timing’ is rated as high or unclear; LOW if all items were rated as low.Pairwise comparison ROB was computed according to ROB judgements for the studies contributing to the direct and indirect meta-analyses. The study percent contribution matrix was calculated using the CINeMA web application which requires a single comparison for each exercise type group within a study, therefore excluded studies that compare only groups of the same exercise type that have other differing design or delivery characteristics. |
| Indirectness | Pairwise comparison indirectness was computed for the studies contributing to the direct and indirect meta-analyses using the CINeMA web application based on individual study ratings. Selection criteria for this review were broad and heterogeneous and all studies were judged to provide relevant evidence. Transitivity was investigated by considering distribution of potential treatment effect modifiers, however, did not modify this assessment due to inconclusive evidence. |
| Incoherence | We assessed incoherence using the approach recommended by CINeMA, which estimates an inconsistency factor with 95% interval (ratio of direct and indirect estimates; no concerns about incoherence if the p-value from SIDE >0.10) and assesses the difference between direct and indirect estimates considering the range of equivalence based on a clinically important difference. |
| Heterogeneity | We assessed heterogeneity by comparing the 95% confidence interval of the pairwise NMA estimate with the prediction interval for NMA estimate. We judged heterogeneity following CINeMA recommendations, by assessing how many crossings of the interval with null effect and clinically important value for in opposite direction as point estimate. |
| Imprecision | We considered the treatment effects included in the 95% confidence interval relative to potentially clinically important differences to assess the precision of the NMA estimate for each pairwise comparison. |
| Publication bias | We assessed suspicion of publication bias by observation of patterns of results of small and large studies using Egger’s test for pairwise comparisons with more than 10 studies available. We rated pairwise comparisons as having suspected publication bias if the p-value <=0.05. |

A：Social interaction disorder

Within-study bias：


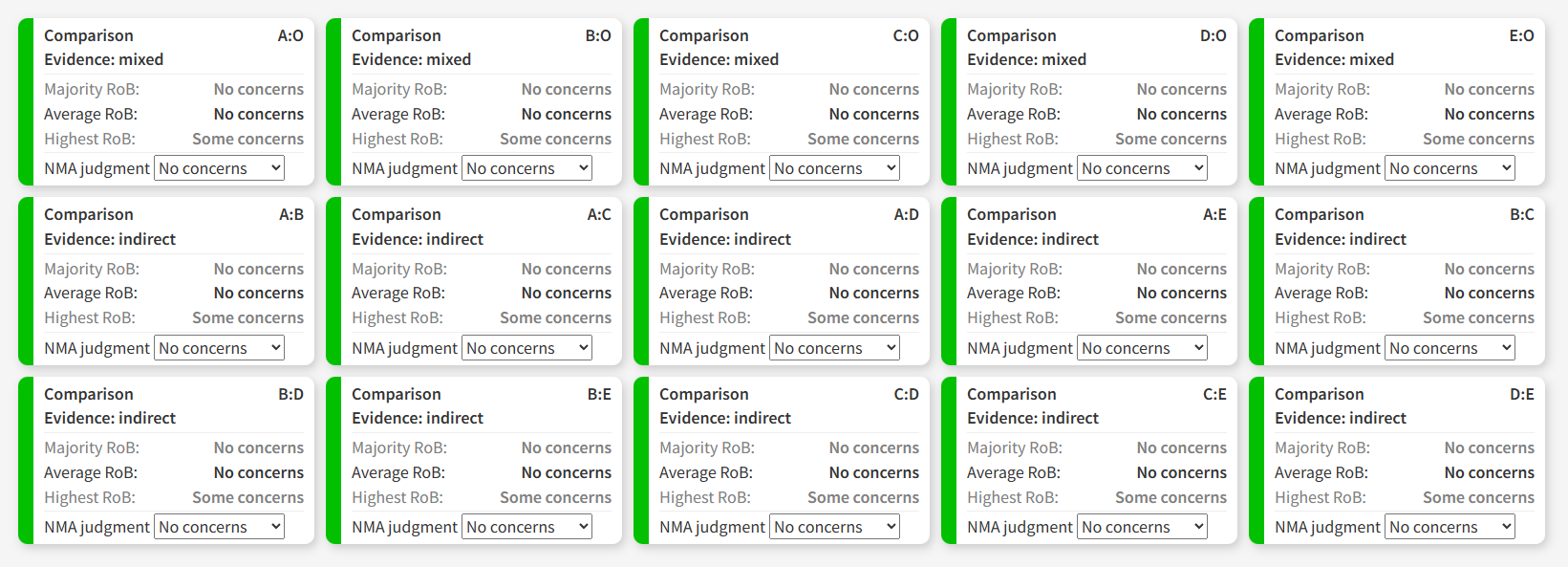


Reporting bias：


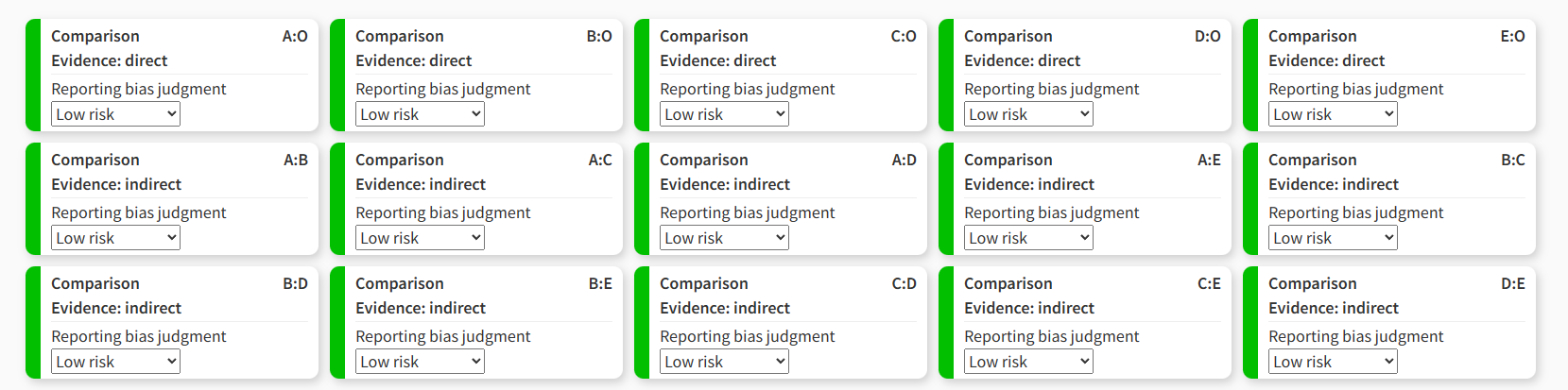


Indirectness：


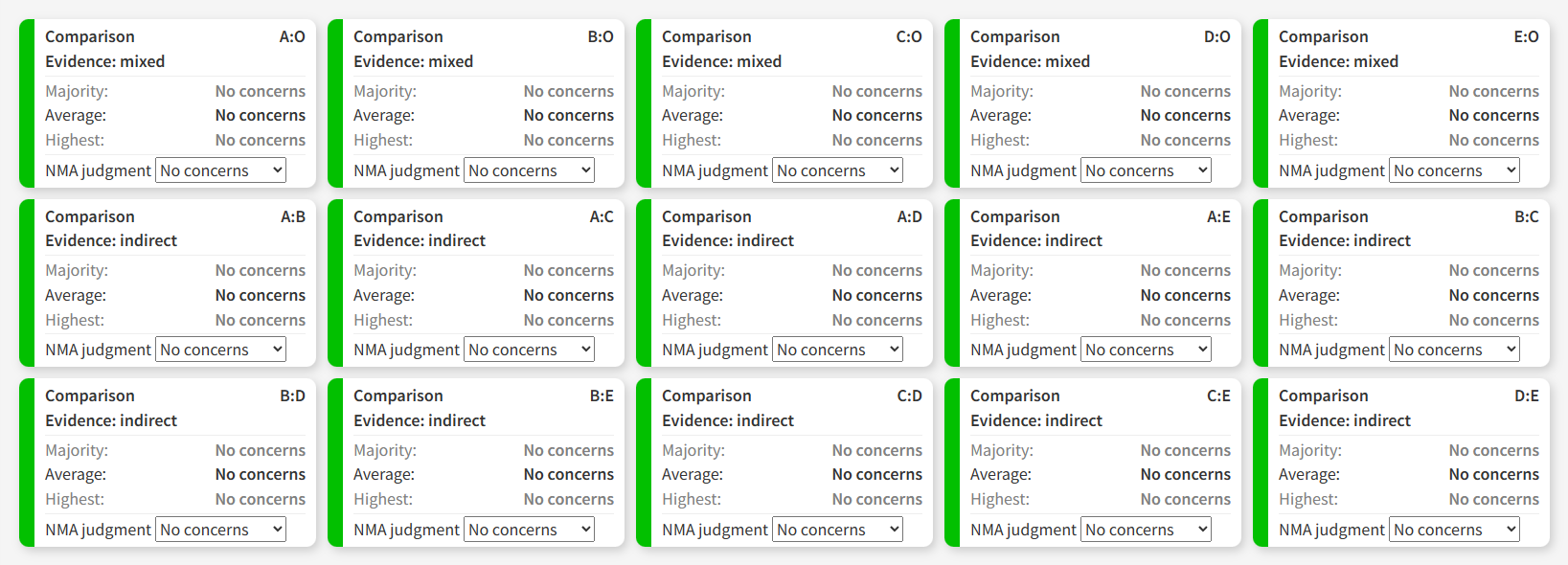


Imprecision：


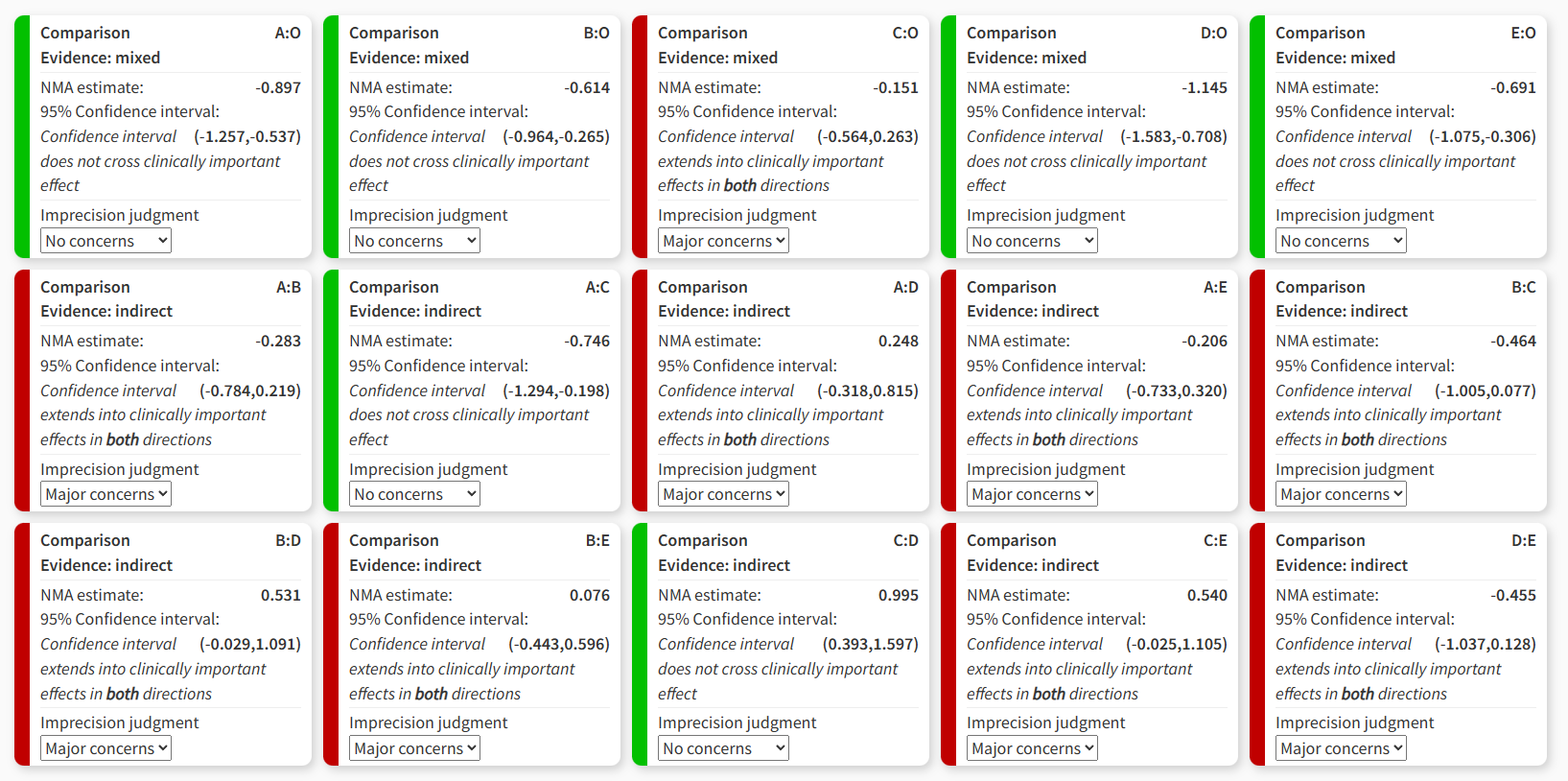


Heterogeneity：


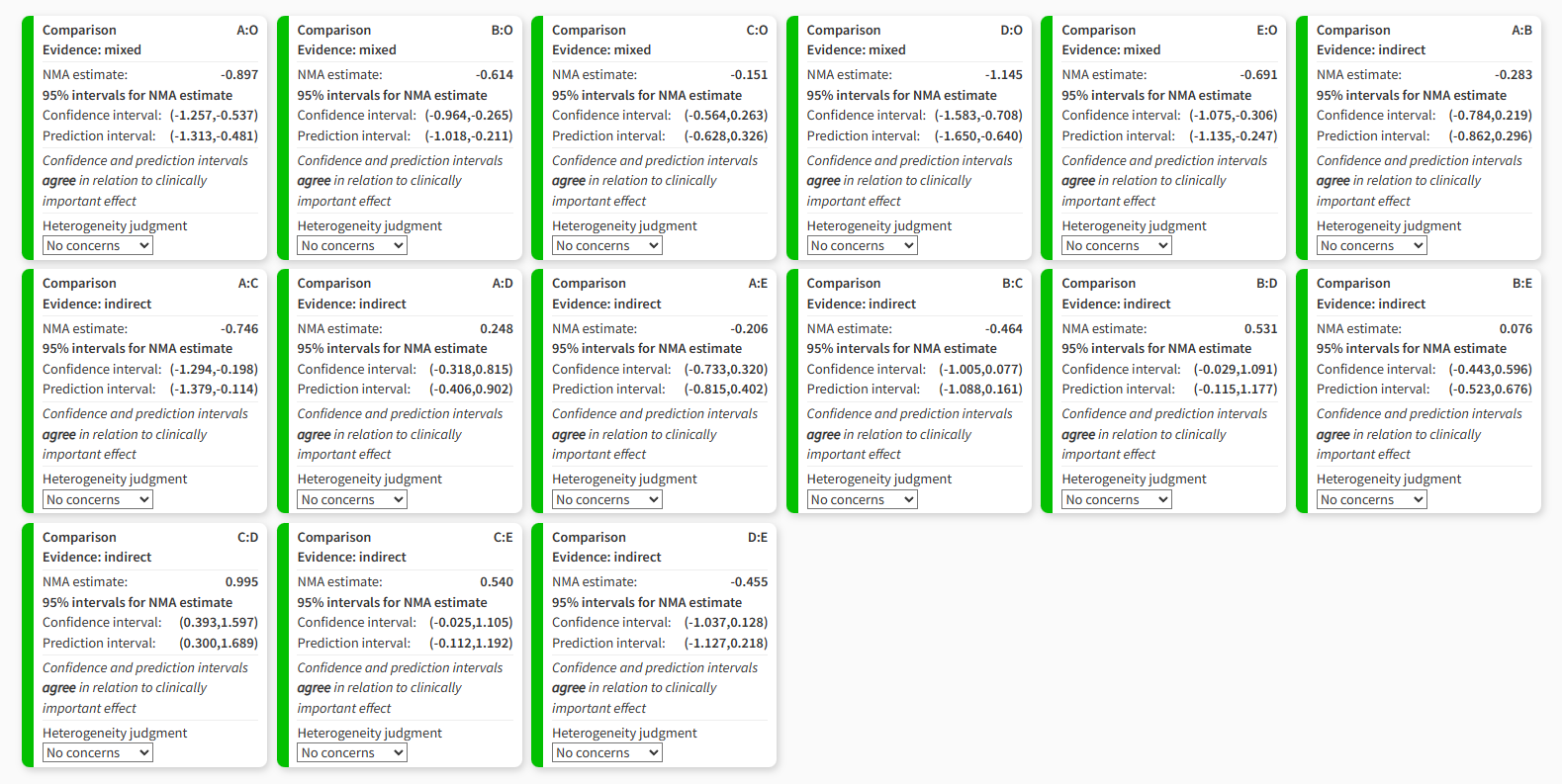


Incoherence：


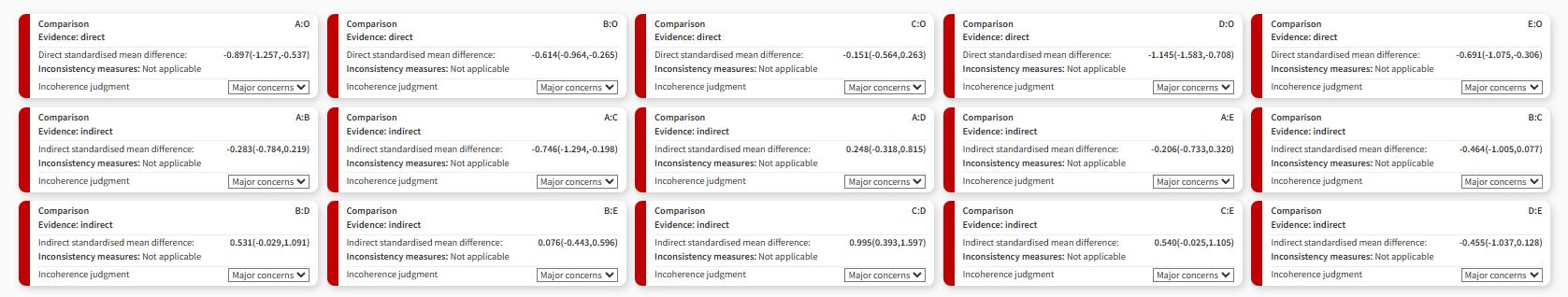


B：Repetition of the stereotyped behavior

Within-study bias：


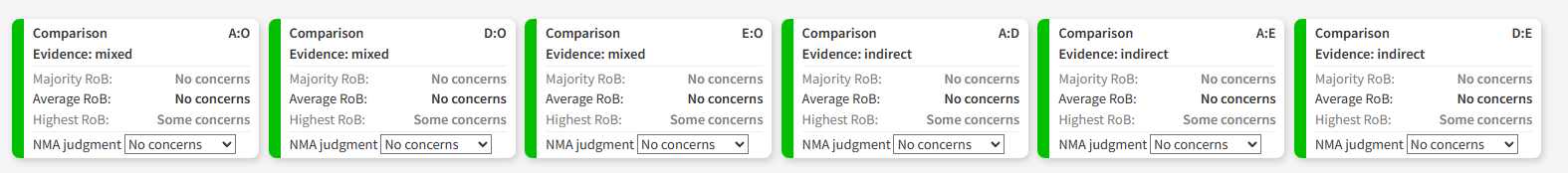


Reporting bias：


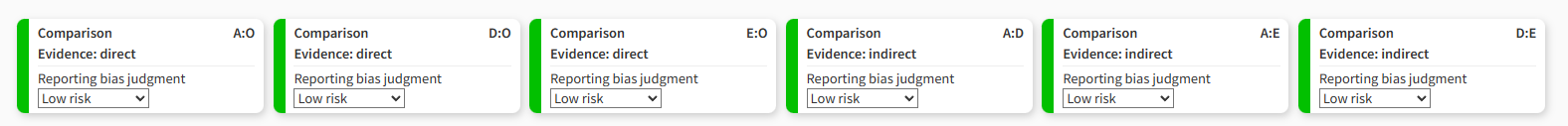


Indirectness：


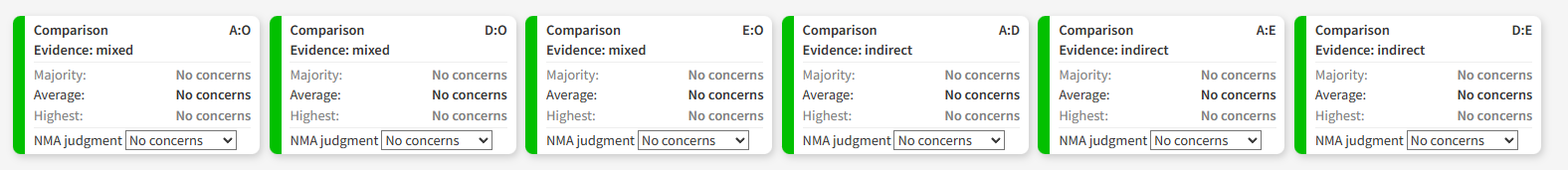


Imprecision：


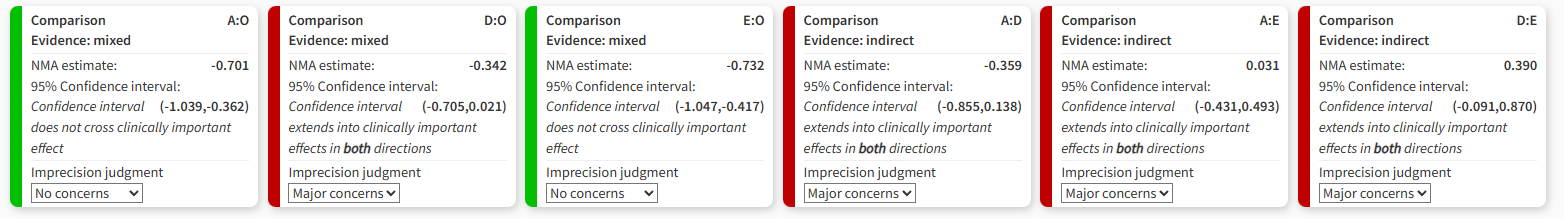


Heterogeneity：


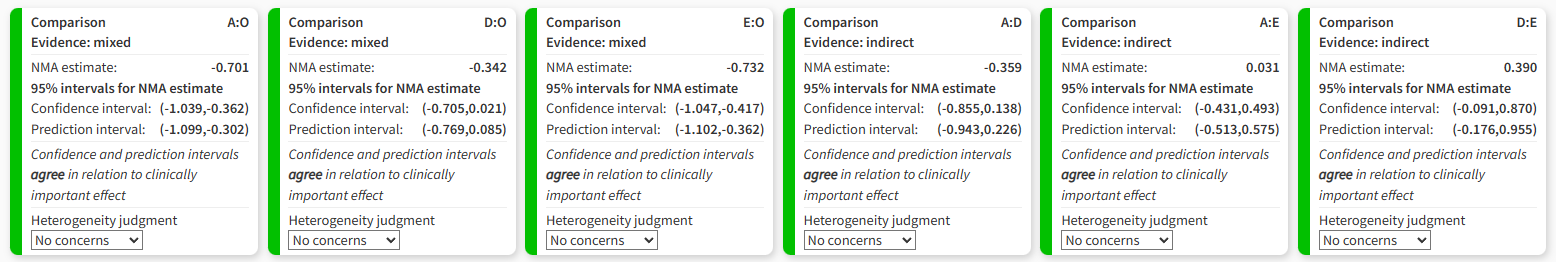


Incoherence：


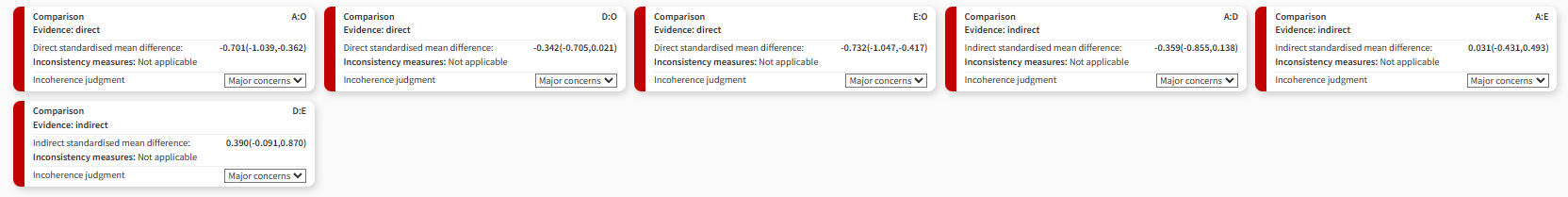


Appendix 7. Citations for 30 included trials.

1. Chan AS, Sze SL, Siu NY, Lau EM, Cheung MC. A chinese mind-body exercise improves self-control of children with autism: a randomized controlled trial. PloS one. 2013;8(7):e68184.
2. Movahedi A, Bahrami F, Marandi SM, Abedi A. Improvement in social dysfunction of children with autism spectrum disorder following long term Kata techniques training. Research in Autism Spectrum Disorders. 2013;7(9):1054-61.
3. Haghighi AH, Broughani S, Askari R, Shahrabadi H, Souza D, Gentil P. Combined Physical Training Strategies Improve Physical Fitness, Behavior, and Social Skills of Autistic Children. Journal of Autism and Developmental Disorders. 2022.
4. Harris A, Williams JM. The Impact of a Horse Riding Intervention on the Social Functioning of Children with Autism Spectrum Disorder. International Journal of Environmental Research and Public Health. 2017;14(7).
5. Tse ACY. Brief Report: impact of a Physical Exercise Intervention on Emotion Regulation and Behavioral Functioning in Children with Autism Spectrum Disorder. Journal of autism and developmental disorders. 2020;50(11):4191‐8.
6. Coman DC, Bass MP, Alessandri M, Ghilain CS, Llabre MM. Effect of Equine Assisted Activities on Social and Sensory Functioning of Children with Autism. Society & Animals. 2018;26(6):551-75.
7. Nekar DM, Lee D-Y, Hong J-H, Kim J-S, Kim S-G, Seo Y-G, et al. Effects of Augmented Reality Game-Based Cognitive-Motor Training on Restricted and Repetitive Behaviors and Executive Function in Patients with Autism Spectrum Disorder. Healthcare. 2022;10(10).
8. Bahrami F, Movahedi A, Marandi SM, Sorensen C. The Effect of Karate Techniques Training on Communication Deficit of Children with Autism Spectrum Disorders. Journal of autism and developmental disorders. 2016;46(3):978‐86.
9. Caputo G, Ippolito G, Mazzotta M, Sentenza L, Muzio MR, Salzano S, et al. Effectiveness of a Multisystem Aquatic Therapy for Children with Autism Spectrum Disorders. Journal of Autism and Developmental Disorders. 2018;48(6):1945-56.
10. Moradi H, Sohrabi M, Taheri H, Khodashenas E, Movahedi A. Comparison of the effects of perceptual-motor exercises, vitamin D supplementation and the combination of these interventions on decreasing stereotypical behavior in children with autism disorder. International Journal of Developmental Disabilities. 2020;66(2):122-32.
11. Marzouki H, Soussi B, Selmi O, Hajji Y, Marsigliante S, Bouhlel E, et al. Effects of Aquatic Training in Children with Autism Spectrum Disorder. Biology-Basel. 2022;11(5).
12. Phung JN, Goldberg WA. Mixed martial arts training improves social skills and lessens problem behaviors in boys with Autism Spectrum Disorder. Research in autism spectrum disorders. 2021;83.
13. Wang JG, Cai KL, Liu ZM, Herold F, Zou L, Zhu LN, et al. Effects of mini-basketball training program on executive functions and core symptoms among preschool children with autism spectrum disorders. Brain sciences. 2020;10(5).
14. Cai K, Yu Q, Herold F, Liu Z, Wang J, Zhu L, et al. Mini-basketball training program improves social communication and white matter integrity in children with autism. Brain sciences. 2020;10(11):1‐14.
15. Cai K-L, Wang J-G, Liu Z-M, Zhu L-N, Xiong X, Klich S, et al. Mini-Basketball Training Program Improves Physical Fitness and Social Communication in Preschool Children with Autism Spectrum Disorders. Journal of Human Kinetics. 2020;73(1):267-78.
16. Hildebrandt MK, Koch SC, Fuchs T. "We Dance and Find Each Other"1: Effects of Dance/Movement Therapy on Negative Symptoms in Autism Spectrum Disorder. Behavioral sciences (Basel, Switzerland). 2016;6(4).
17. Bass MM, Duchowny CA, Llabre MM. The effect of therapeutic horseback riding on social functioning in children with autism. Journal of autism and developmental disorders. 2009;39(9):1261‐7.
18. Zanobini M, Solari S. Effectiveness of the Program "Acqua Mediatrice di Comunicazione" (Water as a Mediator of Communication) on Social Skills, Autistic Behaviors and Aquatic Skills in ASD Children. Journal of Autism and Developmental Disorders. 2019;49(10):4134-46.
19. Gabriels RL, Pan Z, Dechant B, Agnew JA, Brim N, Mesibov G. Randomized Controlled Trial of Therapeutic Horseback Riding in Children and Adolescents With Autism Spectrum Disorder. Journal of the American Academy of Child and Adolescent Psychiatry. 2015;54(7):541‐9.
20. Koch SC, Mehl L, Sobanski E, Sieber M, Fuchs T. Fixing the mirrors: a feasibility study of the effects of dance movement therapy on young adults with autism spectrum disorder. Autism. 2015;19(3):338-50.
21. Yang S, Liu Z, Xiong X, Cai K, Zhu L, Dong X, et al. Effects of mini-basketball training program on social communication impairment and executive control network in preschool children with autism spectrum disorder. International journal of environmental research and public health. 2021;18(10).
22. Aithal S, Karkou V, Makris S, Karaminis T, Powell J. A Dance Movement Psychotherapy Intervention for the Wellbeing of Children With an Autism Spectrum Disorder: A Pilot Intervention Study. Frontiers in psychology. 2021;12:588418.
23. Xu W, Yao J, Liu W. INTERVENTION EFFECT OF SENSORY INTEGRATION TRAINING ON THE BEHAVIORS AND QUALITY OF LIFE OF CHILDREN WITH AUTISM. Psychiatria Danubina. 2019;31(3):340-6.
24. Dong XX, Chen AG, Liu ZM, Wang JG, Cai KL, Xiong X. The effects of basketball on repetitive and stereotyped behaviors and gray matter volume in preschool children with autism. Chinese Sports Science and Technology. 2020;56(11):25-31.
25. Liu RS, Zhan XM, Li XF, Dai L, Zhang SJ, Hong JY, et al. Effects and correlation of gross motor
26. intervention on movement and social skills in children with autism. Chinese School Health. 2021;42(03):358-62+66
27. Song SS, Chai H, Jin PH. The influence of aquatic exercise therapy on social behavior and daily living abilities in children with autism. Chinese Journal of Min K’un Medicine. 2020;32(01):103-5. 39. Wang H, Ma Z. Efficacy analysis of aquatic exercise therapy combined with rehabilitation nursing based on Halliwick technique on autism spectrum disorders. Chinese Journal of Rehabilitation Medicine. 2020;35(9):1108-10.
28. Xiong Y, Yang WH, Ouyang Y, Hu JH. Rehabilitation effects of basic motor skill intervention on movement disorders in children with autism spectrum disorders. Medical Clinical Research. 2021;38(12):1833-6.
29. Yang Y, Yang Y. Analysis of the therapeutic effects of exercise intervention on children with autism. Chinese Journal of Contemporary Medicine. 2016;23(04):29-31.
30. Zhang J, Yang JQ. The effects of sports intervention on behavior and quality of life in children with autism. Chinese Clinical Research. 2017;30(09):1244-6.

Appendix 8. Detailed table of included trials. Description of each included trial including population, setting, exercise and comparison treatments.

| Literature included | Countries | | Sample  E/C | Age（years） | | Intervention type | | Intervention dose | | | Outcome index |
| --- | --- | --- | --- | --- | --- | --- | --- | --- | --- | --- | --- |
|  |  |  |  | Experimental group | Control group | Experimental group | Control group | Period  Week | Frequency  Times/ week | Time  Min/time |  |
| AgnesS.Chan  2013(Chan, Sze, Siu, Lau, & Cheung, 2013) | | China | 20/20 | 11.28±3.90 | 12.42±3.25 | NYG  + Muscle relaxation | Muscle relaxation | 4 | 2 | 60 | ATEC |
| Ahmadreza.M  2013(Movahedi, Bahrami, Marandi, & Abedi, 2013) | | Iran | 13/13 | 9.54±3.43 | 9.06±3.33 | KD | Daily activities | 14 | NR | 90 | GARS-2 |
| AmirHossein.  H2022(Haghighi et al., 2022) | | Iran | 8/8 | 9.00±1.31 | 8.13±1.36 | CPT | No intervention | 8 | NR | 60~70 | GARS-2 |
| Androulla.H  2017(Harris & Williams, 2017) | | Britain | 10/14 | 7.96±0.78 | 6.97±0.33 | THR | No intervention | 7 | NR | 45 | CARS-2/  ABC-C |
| AndyC.Y.Tse  2020(Tse, 2020) | | China | 15/12 | 10.07±1.10 | 9.42±0.90 | CPT | Daily activities | 12 | 4 | 30 | CBCL |
| DrewC.Coman  2017(Coman, Bass, Alessandri, Ghilain, & Llabre, 2018) | | America | 25/25 | 8.70±1.60 | 8.70±1.60 | THR | Daily activities | 12 | 1 | 70 | SRS |
| Daekook M. N  2022(Daekook M. Nekar et al., 2022) | | South Korea | 12/12 | 14.42 ± 5.14 | 14.17 ± 5.09 | CPT  +  Cognitive therapy | Cognitive therapy | 4 | 2 | 30 | RRBs |
| Fatimah.B  2015(Bahrami, Movahedi, Marandi, & Sorensen, 2016) | | Iran | 15/15 | 9.20±3.32 | 9.06±3.33 | KD  +  Educational intervention | Educational intervention | 14 | 4 | 90 | GARS-2 |
| Giovanni.C  2018(Caputo et al., 2018) | | Italy | 13/13 | 8.3±2.30 | 7.7±2.00 | AT  + Conventional treatment | Conventional treatment | 24 | 1 | 45 | CARS/  VABS |
| HadiMoradi  2018(Moradi, Sohrabi, Taheri, Khodashenas, & Movahedi, 2020) | | Iran | 25/25 | 1.03±7.64 | 1.25±7.20 | SIT | Daily activities | 8 | 2 | 50 | GARS-2 |
| Hamza M1  2022(Marzouki et al., 2022) | | Switzerland | 8/6 | 6.3 ± 0.50 | 6.3 ± 0.50 | AT | Daily activities | 8 | 2 | 50 | GARS-2 |
| Hamza M2  2022(Marzouki et al., 2022) | | Switzerland | 8/6 | 6.4 ± 0.50 | 6.3 ± 0.50 | AT | Daily activities | 8 | 2 | 50 | GARS-2 |
| JaniceN.Phung  2021(Phung & Goldberg, 2021) | | America | 14/20 | 9.10±1.10 | 9.52±1.07 | MMAT | Daily activities | 13 | 2 | 45 | SSIS |
| Jin-GuiWang  2020(J. G. Wang et al., 2020) | | China | 18/15 | 5.11±0.65 | 4.70±0.70 | MBTP | Daily activities | 12 | 5 | 40 | SRS-2  /RBS-R |
| KelongCai  2020(K. Cai et al., 2020) | | China | 15/14 | 5.13±0.61 | 4.68±0.72 | MBTP | Daily activities | 12 | 5 | 40 | SRS-2 |
| Ke-LongCai  2020(K.-L. Cai et al., 2020) | | China | 30/29 | 4.56±0.84 | 5.03±0.64 | MBTP | Daily activities | 12 | 5 | 40 | SRS-2 |
| MalinK.H  2016(Hildebrandt, Koch, & Fuchs, 2016) | | Germany | 53/22 | 23.07±8.54 | 21.27±5.32 | DMT | No intervention | 10 | NR | 60 | SANS |
| MargaretM.B  2009(Bass, Duchowny, & Llabre, 2009) | | America | 19/15 | 6.95±1.67 | 7.73±1.65 | THR  + Conventional treatment | Conventional treatment | 12 | 1 | 60 | SRS |
| Mirella.Z.B  2019(Zanobini & Solari, 2019) | | Italy | 13/12 | 5.69±1.27 | 5.42±1.54 | AT  + Conventional treatment | Conventional treatment | 24 | 0.5 | 30 | SRS/ABC |
| RobinL.G  2015(Gabriels et al., 2015) | | America | 58/58 | 10.5±3.20 | 10.0±2.70 | THR | Daily activities | 10 | NR | 45 | SRS/  VABS-II |
| SabineCKoch  2015(Koch, Mehl, Sobanski, Sieber, & Fuchs, 2015) | | Germany | 16/15 | 22.00±7.70 | 22.00±7.70 | DMT | No intervention | 7 | NR | 60 | FBT |
| SixinYang  2021(Yang et al., 2021) | | China | 15/15 | 5.03±0.55 | 4.67±0.70 | MBTP+ Routine rehabilitation | Routine rehabilitation | 12 | 5 | 40 | SRS-2 |
| SuprithaAithal2021(Aithal, Karkou, Makris, Karaminis, & Powell, 2021) | | Britain | 10/16 | 11.53 | 9.77 | DMP  +  Routine nursing | Routine nursing | 5 | 2 | 40 | SCQ |
| WenxinXu  2019(Xu, Yao, & Liu, 2019) | | China | 50/53 | 6.17±2.44 | 6.18±2.94 | SIT  + Conventional treatment | Conventional treatment | 16 | NR | NR | CARS |
| Dong, X2020( Dong, X., & et al., 2020) | | China | 15/15 | 4.67±0.70 | 4.97±0.61 | MBTP  + Routine rehabilitation | Routine rehabilitation | 12 | 5 | 40 | RRBs |
| Liu, R2021( Liu, R., & et al., 2021) | | China | 13/10 | 8.23±1.30 | 8.10±1.37 | CPT | Daily activities | 6 | 4 | 60 | SRS |
| Song, S2020( Song, S., & et al, 2020) | | China | 46/46 | 8.27±0.68 | 8.39±0.53 | AT + Routine nursing | Routine nursing | 16 | 2~3 | 90 | ATEC |
| Wang, H2020( Wang, H., & Ma, Z., 2020) | | China | 28/26 | 7.36±1.65 | 5.6.±1.62 | AT + Routine nursing | Routine nursing | 8 | NR | 90 | ABC |
| Xiong, Y.2021( Xiong, Y., & et al, 2021) | | China | 50/50 | 7.75±1.08 | 8.03±1.97 | CPT  + Conventional treatment | Conventional treatment | 10 | 2 | 60 | ABC |
| Yang, Y.2016( Yang, Y., & Yang, Y., 2016) | | China | 40/40 | 4.90±1.26 | 4.90±1.26 | CPT  + Conventional education | Conventional education | 24 | 6 | 90 | CARS |
| Zhang, J2017( Zhang, J., & Yang, J., 2017) | | China | 30/30 | 7.62±3.14 | 7.54±2.96 | CPT  + Conventional treatment | Conventional treatment | 24 | 7 | NR | CARS |

Appendix 9. Detailed risk of bias assessments for each included trial.


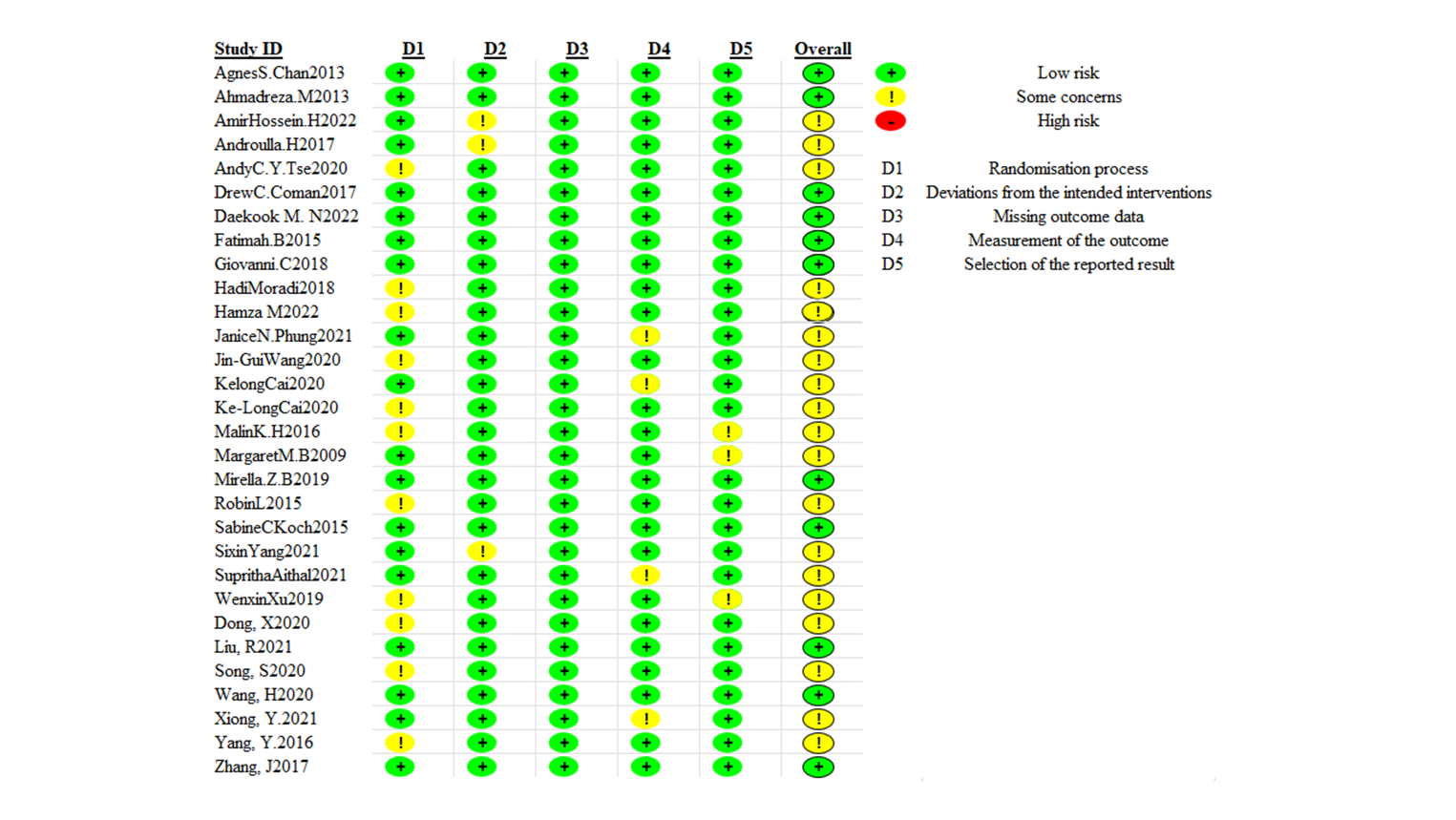


Appendix 10. Sensitivity analysis of included study outcome indicators

A：Social interaction disorder

B：Repetition of the stereotyped behavior

Appendix 11. Judgments for each domain and overall certainty rating for each pairwise comparison (CINeMA assessment). Blue indicates lower-quality evidence; green indicates moderate-quality evidence; shades of colour indicate the magnitude of different risks.

A：Social interaction disorder

| Comparison | Number of studies | Within-study bias | Reporting bias | Indirectness | Imprecision | Heterogeneity | Incoherence | Confidence rating |
| --- | --- | --- | --- | --- | --- | --- | --- | --- |
| A:O | 4 | No concerns | Low risk | No concerns | No concerns | No concerns | Major concerns | Moderate |
| B:O | 3 | No concerns | Low risk | No concerns | No concerns | No concerns | Major concerns | Moderate |
| C:O | 3 | No concerns | Low risk | No concerns | Major concerns | No concerns | Major concerns | Low |
| D:O | 2 | No concerns | Low risk | No concerns | No concerns | No concerns | Major concerns | Moderate |
| E:O | 3 | No concerns | Low risk | No concerns | No concerns | No concerns | Major concerns | Moderate |
| A:B | 0 | No concerns | Low risk | No concerns | Major concerns | No concerns | Major concerns | Low |
| A:C | 0 | No concerns | Low risk | No concerns | No concerns | No concerns | Major concerns | Moderate |
| A:D | 0 | No concerns | Low risk | No concerns | Major concerns | No concerns | Major concerns | Low |
| A:E | 0 | No concerns | Low risk | No concerns | Major concerns | No concerns | Major concerns | Low |
| B:C | 0 | No concerns | Low risk | No concerns | Major concerns | No concerns | Major concerns | Low |
| B:D | 0 | No concerns | Low risk | No concerns | Major concerns | No concerns | Major concerns | Low |
| B:E | 0 | No concerns | Low risk | No concerns | Major concerns | No concerns | Major concerns | Low |
| C:D | 0 | No concerns | Low risk | No concerns | No concerns | No concerns | Major concerns | Moderate |
| C:E | 0 | No concerns | Low risk | No concerns | Major concerns | No concerns | Major concerns | Low |
| D:E | 0 | No concerns | Low risk | No concerns | Major concerns | No concerns | Major concerns | Low |

B：Repetition of the stereotyped behavior

| Comparison | Number of studies | Within-study bias | Reporting bias | Indirectness | Imprecision | Heterogeneity | Incoherence | Confidence rating |
| --- | --- | --- | --- | --- | --- | --- | --- | --- |
| A:O | 4 | No concerns | Low risk | No concerns | No concerns | No concerns | Major concerns | Moderate |
| D:O | 2 | No concerns | Low risk | No concerns | Major concerns | No concerns | Major concerns | Low |
| E:O | 3 | No concerns | Low risk | No concerns | No concerns | No concerns | Major concerns | Moderate |
| A:D | 0 | No concerns | Low risk | No concerns | Major concerns | No concerns | Major concerns | Low |
| A:E | 0 | No concerns | Low risk | No concerns | Major concerns | No concerns | Major concerns | Low |
| D:E | 0 | No concerns | Low risk | No concerns | Major concerns | No concerns | Major concerns | Low |
