# Supplementary material for: A network meta-analysis of the effect of physical exercise on core symptoms in patients with autism spectrum disorders
Source: Front Neurol. 2024 May 9;15:1360434. doi: 10.3389/fneur.2024.1360434 (PMC11113547; doi:10.3389/fneur.2024.1360434)
Supplement: Supplementary file 2 [file Data_Sheet_2.DOCX]

**Supplementary Material 2:** Pearson’s correlation coefficient of the individual items of the Parkinson’s Disease Screening questionnaire

| **Parkinson’s Disease Screening Questionnaire items** | **R** | **P value** |
| --- | --- | --- |
| Q1. Have you ever noticed a tremor of your hands or legs at rest? | 0.763 | < 0.001* |
| Q2. Have you written slower, or your handwriting changed or become smaller? | 0.914 | < 0.001* |
| Q3. Have you notice that you become more clumsy or slower in any tasks: for example, combing, dressing, or bathing or have more difficulty involving fine hand motor control task: for example, doing up your buttons or opening the bottle? | 0.915 | < 0.001* |
| Q4. Have you noticed that your voice has become softer or more monotonous or that listeners have to ask repeatedly because they cannot hear you speak? | 0.871 | < 0.001* |
| Q5. Have you notice that your arm swing less or do not swing when you walk? | 0.848 | < 0.001* |
| Q6. Do you walk with short steps or shuffling gait? | 0.874 | < 0.001* |
| Q7. Do you have difficulty to walk or being freeze while start starting to walk, walking, or turning? | 0.834 | < 0.001* |
| Q8. Do you have trouble with throwing yourself forward while walking causing you to keep up with your step or difficulty to stop walking immediately? | 0.714 | < 0.001* |
| Q9. Do you have any of the following problems? Difficulty turning over when sleeping or getting out of bed or after sitting down. | 0.740 | < 0.001* |
| Q10. Do your tremor, slowness, or stiffness start at one side of your body first? | 0.915 | < 0.001* |
| Q11. Do you know that you have symptoms of speaking or shouting or moving your arms and legs that may be consistent with dreams or fall off the bed while sleeping? Or have you ever been told by a bed partner or caregiver that you have these symptoms? | 0.624 | < 0.001* |
| Q12. Do you usually have excessive sleepiness during the day or fall asleep while doing activity? | 0.633 | < 0.001* |
| Q13. Do you feel that your sense of smell has decreased? | 0.512 | < 0.001* |
| Q14. Do you have chronic constipation, defined by defecating less than 3 times/ week in the past 3 months? | 0.509 | < 0.001* |
| Q15. Do you have symptoms of depression? Crying more easily than usual or lack of interest in the surrounding environment or things that used to be fun in the past. | 0.426 | < 0.001* |
| Q16. Have you ever seen a hallucination or hear a sound without a person? | 0.497 | < 0.001* |
| Q17. Do you usually have lightheaded or dizzy when changing position from supine or sit to stand up, and symptoms usually improve or disappear after sitting or lying down? | 0.374 | < 0.001* |
| Q18. Do you usually have urinary control problems, such as being unable to control urine, urinary incontinence, or urinary retention? | 0.633 | < 0.001* |
| Q19. Do you have problems with analytical thinking, memory, or calculation that has worsened for more than 1 year? | 0.445 | < 0.001* |
| Q20. Do you have balance problems or frequent fall at the very beginning of the tremor, slowness, or stiffness? | 0.734 | < 0.001* |
